# Determinants of Deteriorated Self-Perceived Health Status among Informal Settlement Dwellers in South Africa

**DOI:** 10.3390/ijerph20054174

**Published:** 2023-02-26

**Authors:** Tholang Mokhele, Chipo Mutyambizi, Thabang Manyaapelo, Amukelani Ngobeni, Catherine Ndinda, Charles Hongoro

**Affiliations:** 1 Geospatial Analytics, eResearch Knowledge Centre, Human Sciences Research Council, Pretoria 0001, South Africa; 2 Human Sciences Research Council, Pretoria 0001, South Africa; 3 Africa Health Research Institute, KwaZulu-Natal, Somkhele 3935, South Africa; 4 Human and Social Capabilities Division, Human Sciences Research Council, Cape Town 8000, South Africa; 5 Developmental, Capable and Ethical State Division, Human Sciences Research Council, Pretoria 0001, South Africa

**Keywords:** self-perceived health, informal settlement dwellers, living standard measure, South Africa

## Abstract

Self-perceived health (SPH) is a widely used measure of health amongst individuals that indicates an individual’s overall subjective perception of their physical or mental health status. As rural to urban migration increases, the health of individuals within informal settlements becomes an increasing concern as these people are at high health and safety risk due to poor housing structures, overcrowding, poor sanitation and lack of services. This paper aimed to explore factors related to deteriorated SPH status among informal settlement dwellers in South Africa. This study used data from the first national representative Informal Settlements Survey in South Africa conducted by the Human Sciences Research Council (HSRC) in 2015. Stratified random sampling was applied to select informal settlements and households to participate in the study. Multivariate logistic regression and multinomial logistic regression analyses were performed to assess factors affecting deteriorated SPH among the informal settlement dwellers in South Africa. Informal settlement dwellers aged 30 to 39 years old (OR = 0.332 95%CI [0.131–0.840], *p* < 0.05), those with ZAR 5501 and more household income per month (OR = 0.365 95%CI [0.144–0.922], *p* < 0.05) and those who reported using drugs (OR = 0.069 95%CI [0.020–0.240], *p* < 0.001) were significantly less likely to believe that their SPH status had deteriorated compared to the year preceding the survey than their counterparts. Those who reported always running out of food (OR = 3.120 95%CI [1.258–7.737], *p* < 0.05) and those who reported having suffered from illness or injury in the past month preceding the survey (OR = 3.645 95%CI [2.147–6.186], *p* < 0.001) were significantly more likely to believe that their SPH status had deteriorated compared to the year preceding the survey than their counterparts. In addition, those who were employed were significantly (OR = 1.830 95%CI [1.001–3.347], *p* = 0.05) more likely to believe that their SPH status had deteriorated compared to the year preceding the survey than those who were unemployed with neutral SPH as a base category. Overall, the results from this study point to the importance of age, employment, income, lack of food, drug use and injury or illness as key determinants of SPH amongst informal settlement dwellers in South Africa. Given the rapid increasing number of informal settlements in the country, our findings do have implications for better understanding the drivers of deteriorating health in informal settlements. It is therefore recommended that these key factors be incorporated into future planning and policy development aimed at improving the standard of living and health of these vulnerable residents.

## 1. Introduction

Rural–urban migration in Africa and South Africa, in particular, is a key contributor to the increase in people living in informal settlements. Whilst moving to these urban settlements holds the promise of a better lifestyle and economic opportunities, urban informal settlements in South Africa are often characterised by overcrowding, safety issues, unemployment, hunger, poor basic services delivery and inequalities [1,2,3,4]. The risks imposed by physical housing structures and living environments in informal settlements have considerable impacts on the health and well-being of these vulnerable groups, potentially exposing them to various diseases [2,5,6] and making them especially vulnerable during pandemics such as the COVID-19 pandemic [7,8]. It is anticipated that the implementation of Universal Health Coverage in South Africa, namely National Health Insurance (NHI), will have positive effects on the health of these informal settlement dwellers. For instance, it was reported that the Health Transformation Plan (HTP) had good effects on the health level of informal settlement residents in Iran by ensuring that they had insurance coverage and reducing many economic, social as well as cultural problems, with reduced out-of-pocket expenditures [9]. Previous studies show that informal settlement dwellers are more likely to self-report ill health and, due to the spatial and social marginalisation, are at an increased risk of experiencing mental health issues [7,10,11]. These vulnerable communities in informal settlements often find themselves further marginalised through labour policies that are not designed to accommodate them [8].

Self-perceived health (SPH), also commonly called self-reported health, self-rated health or self-assessed health, is a widely used and acceptable measure of health across individuals that has been applied both in international and South African studies [11]. Various studies have validated it as a good measure of health that is consistent with objective measures of health [11] and also as a strong predictor of mortality [12,13], morbidity [13,14] and healthcare use [15]. The World Health Organization (WHO) recognises it as one of the best measures of health [16]. SPH does not focus on one specific dimension of health, but rather it is used as an indicator of an individual’s overall subjective perception of their physical or mental health status. Thus, the presence of any health condition is a predictor of self-perception of health [17,18]. SPH is commonly measured using a single item health measure on a three- or five-point scale ranging from good to bad. Options can take the form of “very good, good, fair, bad, very bad”. Using this scale, individuals are then required to rate their health.

The factors that influence SPH include health-related predictors, clinically diagnosed health status, history of chronic illnesses, lifestyle factors, socio-economic status and social factors [19,20,21,22]. Studies have described health status in relation to living environments within informal settlements in South Africa [2,3,4,6,10,23,24,25]. These studies show that a majority of informal settlement dwellers suffer a disproportionate burden of sickness and disease. Studies that have assessed the determinants of health in poor urban communities in South Africa have focused on a specific disease or a specific community [26,27,28].

There are some studies that have been undertaken to explore factors affecting poor SPH, even though some were not focused on informal settlements. For instance, Kasenda et al. [29] investigated the prevalence of poor SPH and its determinants among 962 participants in Malawi. Kasenda et al. [29] found that poor SPH was associated with being female, increasing age, decreasing education, frequent health care attendance as well as living with disability. Kasenda et al. [29] further reported that prevalence of poor SPH in Malawi was in line with findings from other countries.

Mlangeni et al. [30] explored factors associated with poor SPH amongst individuals from KwaZulu-Natal using data from the 2012 South African national household survey. Mlangeni et al. [30] reported that fair/poor SPH was significantly associated with being older, HIV-positive, being an excessive drinker, being educated, being employed and not accessing care regularly. Mlangeni et al. [30] recommended that education, job opportunities, social services for poor living conditions and poor well-being, provision of health insurance as well as incorporating health promotion initiatives as part of social support and public services for substance abusers should be considered.

Patterson et al. [31] assessed self-rated physical health and related factors in youth residing in slums or informal settlements in Uganda. Patterson et al. [31] found that poor self-rated physical health was significantly associated with older age, lower education, having been injured due to their drinking and having initiated alcohol use early, among others. Patterson et al. [31] further indicated that poor living conditions in the slums are exacerbated by a range of health concerns and risk behaviours, which impact youth’s physical health, which can adversely impact their long-term health and longevity if no interventions are undertaken.

To the best of our knowledge, no nationally representative study has assessed the factors associated with SPH in informal settlements in South Africa, let alone deteriorated or poor SPH. The evaluation of factors associated with SPH in the context of living environments is essential for the design of strategies to improve health.

This paper aims to expand on the existing body of literature on health in South African informal settlements by exploring the factors related to deteriorated SPH status among informal settlement dwellers in South Africa. The need to address these issues is entrenched in the United Nations Sustainable Development Goals (SGDs)—a set of internationally agreed goals and targets for sustainable development by 2030. SDG 3, which targets good health and well-being, can only be met through strategies that include informal settlements [32]. For SDG 3 to be met, living conditions need to be addressed as set out in SDG 11, which seeks to make cities inclusive, safe, resilient and sustainable. A study of this nature is also important because there is a lack of longitudinal studies that assess the impact of informal settlement upgrading or informal settlement housing and basic infrastructural service improvements on health in South Africa [2]. As the study focuses on informal settlements targeted for upgrades, it forms the basis for future studies that seek to explore the health benefits of these settlement upgrades. Furthermore, with the continued growth of informal settlements, it is important to assess the factors that influence SPH. Findings from this study could provide a narrative for policies and interventions targeted at improving population health in informal settlements.

## 2. Materials and Methods

### 2.1. Data

**Figure 1:**
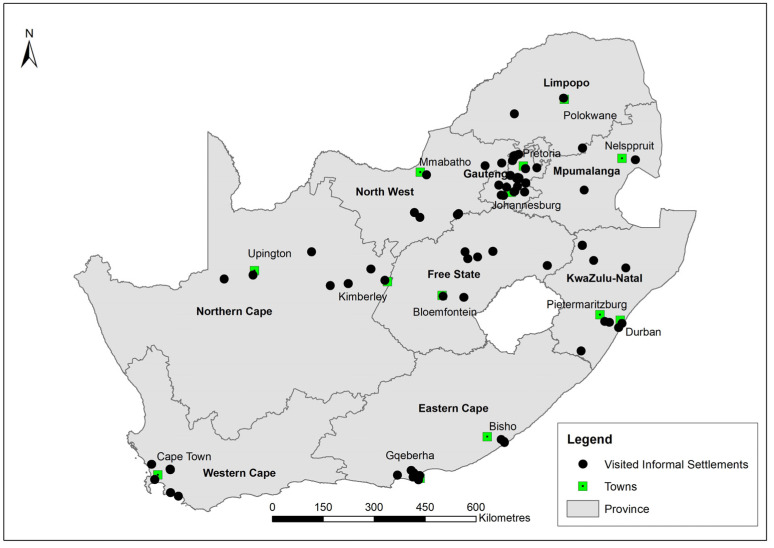
Visited informal settlements across the country.

**Figure A1:**
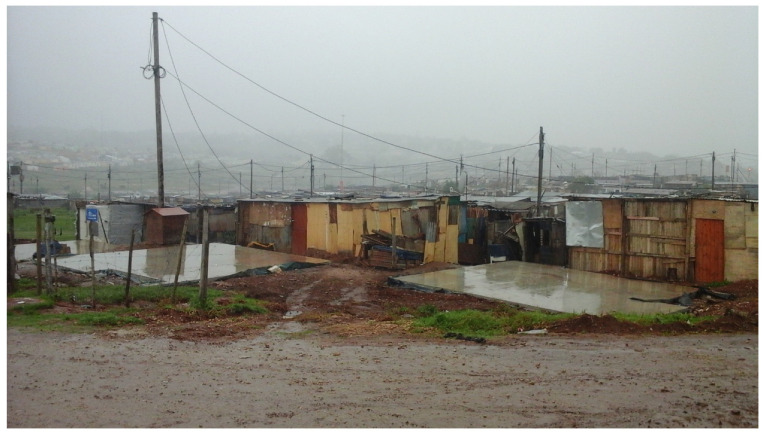
Joe Slovo informal settlement in 2015, Eastern Cape, South Africa.

**Figure A2:**
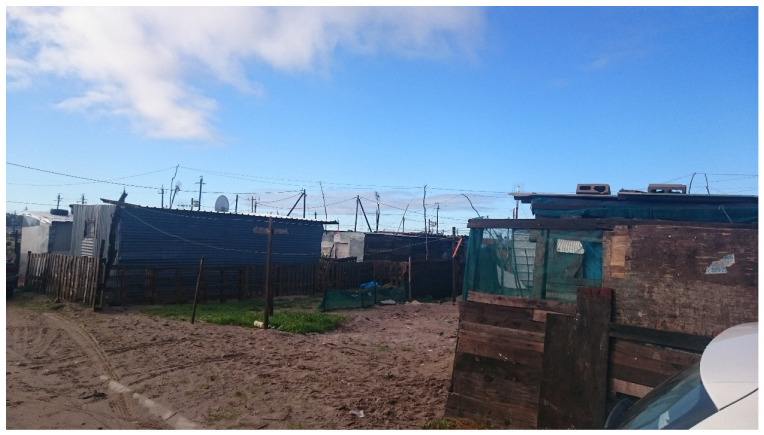
Atlantis Witsand informal settlement in 2015, Western Cape, South Africa.

**Figure A3:**
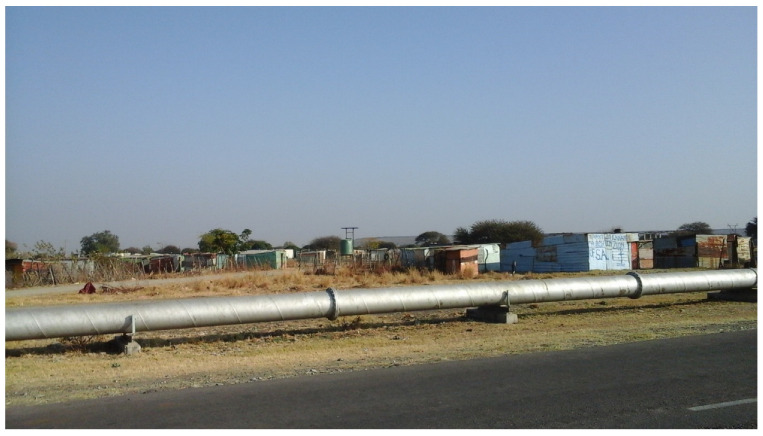
Bokamoso informal settlement in 2015, North West, South Africa.

**Figure A4:**
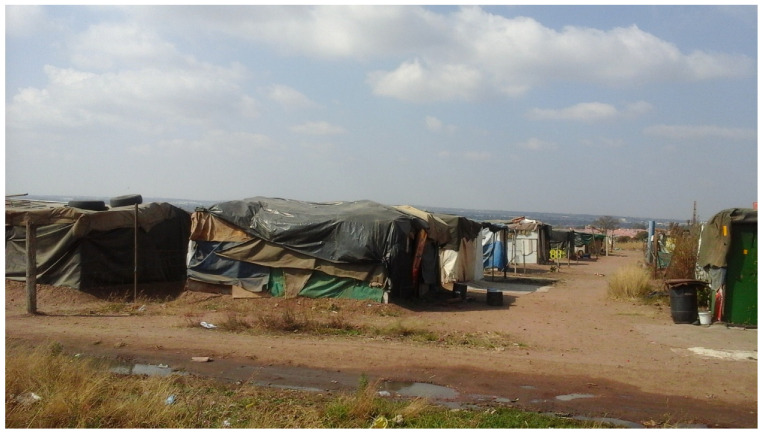
Mohlakaneng Ext106 informal settlement in 2015, Limpopo, South Africa.

This paper used data from the first national representative Informal Settlements Survey in South Africa conducted by the Human Sciences Research Council (HSRC) in 2015. For more details on the methods employed in the survey, please see Ndinda et al. [33]. Briefly, a stratified random sampling method was employed. The total number of informal settlements targeted for upgrading per province was recorded. This was used as an informal settlement sapling frame. The total number of informal settlements differed by province and only 10% were sampled in each province. The number of households in each of the visited informal settlements across the country was generated using satellite imagery. This number of households per informal settlement was used as the sampling frame for household sampling. The total number of households differed by informal settlement and only a fixed number of 45 households were sampled in each informal settlement. This means that both informal settlements and households did not have equal chance of being sampled or selected. The data were weighted to correct this potential bias due to unequal sampling probabilities as well as in order to have a national representative of informal settlements targeted for upgrading in South Africa. The weights were applied using the realised sample in both cases, that is, visited informal settlements and interviewed households. A total of 75 informal settlements were successfully visited across the country (Figure 1). See Appendix A (Figure A1, Figure A2, Figure A3 and Figure A4) for some visual materials about the informal settlements. About 2380 household heads were interviewed using a semi-structured household questionnaire from these informal settlements. The informal settlement weight was calculated as the inverse of the probability of the informal settlement being realised in a province, while the household weight was calculated as the inverse of the probability of the household being interviewed in an informal settlement. The final weight was the product of informal settlement weight and household weight.

A paper-based semi-structured household questionnaire was used for collection of the data and was administered by research assistants. The household questionnaire consisted of geographic particulars, household roster (demographics, education and economic activity of household members), living standard measure, health and nutrition, housing and tenure, access to services and crime and safety (Supplementary File [questionnaire] attached).

In terms of exclusion and inclusion criteria, although a total of 2380 household respondents were interviewed in the whole survey, only 2242 respondents responded to the main outcome question, which asked about how their health was compared with one year prior to their taking the survey. Therefore, the final sample size that was considered for analysis for this paper was 2242. This is due to the fact that respondents were allowed to answer questions that they were willing to answer and they were told of their rights to not answer questions that they were not willing to answer.

### 2.2. Measures

For the outcome variable, the SPH was considered. Respondents were asked how their health was compared with one year prior to their taking the survey with response options being: 1 = somewhat better, 2 = much better, 3 = about the same, 4 = much worse and 5 = somewhat worse. These options were further dichotomised into two: 1 = worse or deteriorated (much worse and somewhat worse) and 0 = better/about the same (somewhat better, much better and about the same) for multivariate logistic regression analysis. The reason behind dichotomising the outcome variable and using multivariate logistic regression was that this study focused on determinants of deteriorated SPH other than general SPH. A similar practice was noticed where the focus was on one aspect of SPH in previous studies [29,30,31,34,35,36,37]. For consideration of ordered regression logistic regression, the outcome variable was categorised into three groups: much worse or deteriorated (much worse and somewhat worse), neutral/about the same (about the same) and better/improved (somewhat better and much better).

Explanatory variables included demographic factors such as sex (male or female), age (18–29, 30–39, 40–49, 50–59 and 60+) and marital status (married/cohabiting, divorced/widowed/separated and single/never married). Socioeconomic factors included education (no/primary school, secondary school and matric/higher), employment (unemployed or employed), household income per month (ZAR 0-ZAR 2000, ZAR 2001-ZAR 5500 and ZAR 5501 and more), whether the household has ever run out of food (yes or no) and Living Standard Measure (low, medium and high). Living Standard Measure was developed using Multiple Correspondence Analysis (MCA). The following 19 asset variables with yes response *n* > 100 were considered from 35 assets: fridge, deep freezer, VCR/DVD, cell phone, washing machine, internet access, electric/gas stove without oven, TV, radio, HI-FI, microwave oven MNET/DSTV, car, iron, electric/gas stove with oven, fan, mattress, bicycle and tools (see Appendix B). All asset variables were coded 0 = no and 1 = yes. Health-related and behavioural factors included illness or injury suffered in the past month prior to taking the survey (yes/no), tobacco use (yes/no), alcohol use (yes/no) and drug use (yes/no).

### 2.3. Data Analysis

Data were analysed in Stata version 15.0 [38]. As indicated, the data were weighted to correct potential bias due to unequal sampling probabilities and to be able to generalise findings to a national representative of informal settlements targeted for upgrading in South Africa. The Stata “svy” command was used to incorporate these weights during data analysis. Differences in categorical variables were compared using Chi-square tests. Multivariate logistic regression analysis was performed to assess factors affecting deteriorated SPH among the informal settlement dwellers in South Africa. Furthermore, ordered regression logistic regression was considered to attain a better understanding of factors associated with deteriorated/worse SPH compared to the other two groups classified as neutral/about the same and better/improved separately, unlike in the case of the multivariate logistic regression wherein the two were grouped together. The Stata “omodel” command was performed to test the proportional odds assumption, and the results revealed that the proportional odds assumption was violated. Multinomial logistic regression analysis, which has been used for ordered outcome variables in previous studies [39,40,41,42], was therefore considered for further analysis. As the focus of this study was on determinants of deteriorated SPH, the two models were run with better/improved SPH being used as base category in the first model while the neutral SPH was the base category in the second model. Odds Ratios (ORs) were reported from the multivariate logistic regression and multinomial logistic regression. Confidence Intervals (CIs) were set at 95%, with a *p* value ≤ 0.05 considered statistically significant in all analyses.

## 3. Results

### 3.1. Background Characteristics of Respondents

The study sample used for this paper consisted of 2242 respondents. Males constituted 54.5% of the sample while females accounted for 45.5% (Table 1). There was no significant difference between males and females with *p* = 0.489. The dominant age group was those aged 30 to 39 years-old at about 30%, followed by those aged 40 to 49 years-old at 26.1%. In terms of marital status, just below half (48.1%) were married or cohabiting, followed by 43.8% who were single or never married. No/primary school and secondary school accounted for around 37% each. The majority of respondents, at 58.9%, fell under the ZAR 0 to ZAR 2000 household income band. Almost one third (31.6%) of the informal settlement dwellers were smokers.

### 3.2. Deteriorated SPH Status among Informal Settlement Dwellers

Table 2 highlights deteriorated SPH and explanatory factors among informal settlement dwellers across the country. Deteriorated SPH status was significantly higher among those with no/primary school (19.6%) and those who did not use drugs (15.3%) compared to their relevant counterparts. Informal settlement dwellers who never ran out of food (10.0%) and those who did not experience illness or injury in the past month prior to taking the survey (11.4%) were significantly less likely to believe that their SPH deteriorated compared to the year prior to taking the survey of their relevant counterparts.

### 3.3. Factors Influencing Deteriorated SPH Status among Informal Settlement Dwellers

Informal settlement dwellers aged 30 to 39 years-old were significantly less (OR = 0.332 95%CI [0.131–0.840], *p* < 0.05) likely to believe that their SPH status had deteriorated compared to the year preceding the survey than those aged 18 to 29 years-old (Table 3). Informal settlement dwellers with ZAR 5501 and more household income were significantly less (OR = 0.365 95%CI [0.144–0.922], *p* < 0.05) likely to believe that their SPH status had deteriorated compared to the year preceding the survey than those in the ZAR 0 to ZAR 2000 household income band. Those who reported always running out of food were significantly more (OR = 3.120 95%CI [1.258–7.737], *p* < 0.05) likely to believe that their SPH status had deteriorated compared to the year preceding the survey than those who never ran out of food. Residents who reported having suffered from illness or injury in the past month preceding the survey were significantly more (OR = 3.645 95%CI [2.147–6.186], *p* < 0.001) likely to believe that their SPH status had deteriorated compared to the year preceding the survey than those who did not. Those who reported using drugs were significantly less (OR = 0.069 95%CI [0.020–0.240], *p* < 0.001) likely to believe that their SPH status had deteriorated compared to the year preceding the survey than those who did not use drugs.

Furthermore, multinomial logistic regression models showed that similar factors (age, ran out of food, injury or illness and drug use) were significantly associated with deteriorated SPH status among informal settlement dwellers, as was the case with multivariate logistic regression analysis (Table 4). The only difference is that household income was not significant in multinomial logistic regression models, and instead, employment was significant when neutral SPH was used as a base category. For instance, employed residents were significantly (OR = 1.830 95%CI [1.001–3.347], *p* = 0.05) more likely to believe that their SPH status had deteriorated compared to the year preceding the survey than those who were unemployed with neutral SPH as a base category.

## 4. Discussion

This paper’s aim was to investigate factors related to deteriorated SPH status among informal settlement residents in a national survey conducted in 2015 in South Africa. This study found that informal settlement residents within a certain age range (between 30 and 39 years), higher income bracket (>R5501) and demonstrating previous use of drugs were significantly less likely to report that their SPH had deteriorated compared to the previous year than their respective counterparts.

Age has been found to be associated with SPH in previous studies [43,44]. This association between age and SPH is not consistent across all studies in the sense that the age ranges associated with SPH varies in different studies. For example, those aged 85 years and older were found to have higher SPH than those aged 64 to 75 years in one study [45], while other studies found no significant differences in SPH between those aged 75 and older and those aged between 35 and 44 years [46], and other studies generally found similarities in SPH across different age subgroups [22]. Bonner et al. [30] found that between 75% and 86% of those aged 40 years and older reported good health. Most participants in this study fell between 30 and 49 years old at 55.9% of the total number. This relatively younger cohort might partly explain the significant perception that SPH had not deteriorated.

Contrary to the findings of this study that residents that were employed were significantly more likely to report deteriorated SPH, Chola and Alaba [34] found that those employed were significantly more likely to report good SPH, while Mlangeni [30] also found those employed were significantly less likely to have fair/poor SPH compared to those who were unemployed. This finding might be caused by the fact that informal settlement residents are predominantly poor, so even those that are employed might be earning less, hence they are not far apart in terms of better wealth compared to their unemployed counterparts. However, this finding needs to be explored further as it is commonly known that poor residents who are unemployed are more likely report poor SPH, especially in the informal settlement setting.

A higher income being associated with perceptions that health status had not deteriorated is consistent with previous studies. Research has shown that negative perceptions of environmental hazards were associated with poor self-perception in a low-income community [46]. Moreover, factors such as lower socio-economic status, living in slums, living in a low-income household and poverty were also associated with poor self-rated health [47,48]. Higher income seems to have had a protective effect against poor SPH status.

The finding of those who reported using drugs having perceptions that health status had not deteriorated is inconsistent with what is found in the literature. Previous studies reported that the more drugs a person used, the greater is the likelihood of reporting poor SPH. In certain instances, users of opioids were found to have poorer self-rated health than other drug users [49], and those who frequently used drugs to cope had higher odds of reporting to be poor SPH [50]. A possible explanation for those who reported using drugs in our study having perceptions that their SPH status had not deteriorated could be perhaps they had consumed drugs at the time of the interview. This inebriated state would have been useful to mask the actual perceptions. In addition, a very small number of informal residents who indicated they used drugs reported that their SPH had deteriorated compared to the previous year preceding the survey. Therefore, this could also contribute to the inconsistent findings of this paper.

Those who reported running out of food and those who had suffered from illness or injury in the past month were more likely to believe that their SPH status had deteriorated compared to the preceding year. People who are diagnosed to have clinical evidence of ill health or those who report morbidity are generally more likely to report poor SPH [51,52]. Poor SPH has also been shown to be associated with frailty and prefrailty in urban-living older adults [53]. The evidence suggests that factors which are more immediate and personal to the individual, such as if they are currently living with an ailment or not or if they are on any treatment, have a significant impact on the overall perception of wellbeing.

SPH should be viewed as reflecting people’s lived experiences, their perceptions of health, access to healthcare and how these interact with lifestyle factors, and should also include biological factors such as sex [54]. This means that a more holistic view of health will have to be adopted, since it has been shown that people who live in informal settlements are constantly navigating structural constraints imposed by lack of access to amenities. More specifically, the state of the informal settlements earmarked for upgrading sampled in this study were characterized by a lack of basic services wherein as much as 52% did not have access to electricity, 55% used communal taps and 53% used pit latrines [42]. This state of lack is likely to lead to distress and low self-esteem, which have been shown to be negatively associated with good health [22]. Therefore, when reporting on SPH, it is important to include variables that characterize and seek to incorporate both the physical and social environments [55].

The foremost goal of conducting this kind of research is to identify vulnerable groups and all the possible ways through which individuals and communities experience poor health [54]. The findings in this study identify some of the specific factors that can be targeted in designing interventions to improve the wellbeing of informal settlement residents in South Africa. These factors can be broadly categorized as structural (higher income, employment and running out of food) and individual (age, use of drugs and injury and illness) to help with the development of these interventions. The findings from this study also provide an overview of the general health conditions of residents of informal settlements targeted for upgrading in South Africa. It is therefore recommended that these key factors be incorporated into future planning and policy development aimed at improving the standard of living and health of these vulnerable residents. In addition, based on the findings of this research, the authors recommend that deteriorated or poor SPH should be considered as an indicator for poor health status especially where physical health examination is not financially feasible. Since the urban poor also make up the majority of the labour in the cities, labour legislation that makes provision for decent housing could help alleviate the structural and environmental influences on ill health and poor SPH [8].

Among the limitations of this study, it is important to bear in mind that SPH is subject to both recall bias and social desirability bias. However, the social desirability bias could have been likely mitigated by the need for improved services, thus generating a higher likelihood of more accurate responses. The people who participated are skewed towards unemployed and lower income groups; therefore, overestimating poor SPH is highly possible. However, the findings from this study provide a general picture of deteriorated SPH and related factors among informal settlements in South Africa.

## 5. Conclusions

SPH is a widely used and validated measure of health that is applied in various literatures. This study contributes to the existing body of the literature on health in South African informal settlements by providing insight into the factors associated with deteriorated SPH status amongst informal settlement dwellers in South Africa. Informal settlement dwellers aged 30 to 39 years old, those with ZAR 5501 and more household income and those who reported using drugs were significantly less likely to believe that their SPH status had deteriorated compared to the year preceding the survey. Those who were employed, reported always running out of food and residents who reported having suffered from illness or injury in the past month preceding the survey were more likely to believe that their SPH status had deteriorated compared to the year preceding the survey. Given the rapidly increasing number of informal settlements across the country, especially in the metropolitan areas such as in Gauteng, Western Cape and KwaZulu-Natal, the evidence provided in this study is important for the development of interventions that work towards health improvement, such as health promotion and treatment programmes that aim to reduce illness and injury. It is therefore recommended that these key factors be incorporated into future planning and policy development aimed at improving the standard of living and health of these vulnerable residents. It is also recommended that deteriorated or poor SPH should be considered as another form of assessment of poor health status among informal settlement residents especially where regular physical health examinations are not possible.

## Figures and Tables

**Table 1:** Characteristics of the study sample.

	Sample	%	95%CI	*p* Value
Total	2242	100		
Demographic factors				
Sex				
Male	1095	54.5	[47.8–61.0]	0.489
Female	1018	45.5	[39.0–52.2]	
Age group				
18 to 29	305	14.6	[11.0–19.1]	0.066
30 to 39	600	29.8	[25.4–34.7]	
40 to 49	551	26.1	[23.5–28.9]	
50 to 59	373	18.4	[14.5–23.0]	
60+	277	11.1	[7.5–16.3]	
Marital status				
Married/cohabiting	1049	48.1	[44.6–51.6]	0.191
Widowed/divorced/separated	250	8.1	[6.2–10.4]	
Single/never married	895	43.8	[40.0–47.8]	
Socio-economic factors				
Education level				
No/Primary school	871	36.9	[33.4–40.6]	0.03
Secondary school	823	36.7	[33.3–40.3]	
Matric/Higher	507	26.4	[22.9–30.1]	
Employment status				
Unemployed	1346	60	[56.1–63.8]	0.623
Employed	853	40	[36.2–43.9]	
Household income				
R0-R2 000	1138	58.9	[50.0–67.3]	0.389
R2 001-R5 500	716	32.8	[26.3–40.0]	
R5 501 and more	149	8.3	[6.2–11.0]	
Ever ran out of food				
Never	648	31.8	[27.3–36.6]	0.009
Sometimes	1271	52.9	[49.6–56.2]	
Always	281	15.3	[10.0–22.8]	
Living Standard Measure				
Low	723	32.1	[23.8–41.7]	0.131
Medium	725	34.3	[31.0–37.8]	
High	783	33.6	[25.5–42.7]	
Health and behavioural factors				
Illness or injury				
No	1663	77.6	[20.2–24.8]	<0.001
Yes	536	22.4	[75.2–79.8]	
Smoke				
No	1397	68.4	[65.2–71.5]	0.321
Yes	813	31.6	[28.5–34.8]	
Alcohol use				
No	1441	69.2	[66.9–71.4]	0.644
Yes	754	30.8	[28.6–33.1]	
Drug use				
No	2046	95.5	[94.3–96.5]	<0.001
Yes	109	4.5	[3.5–5.7]	

CI = Confidence Interval. Subtotals are not always equal to the overall total due to non-response or missing data.

**Table 2:** Respondents’ deteriorated SPH and explanatory factors.

	Sample	%(n)	95%CI	*p* Value
Demographic factors				
Sex				
Male	1095	13.9(152)	[10.5–18.1]	0.494
Female	1018	15.8(161)	[12.2–20.1]	
Age group				
18 to 29	305	13.5(41)	[7.3–23.5]	0.096
30 to 39	600	10.6(64)	[7.2–15.4]	
40 to 49	551	14.7(81)	[10.1–20.9]	
50 to 59	373	21.3(79)	[14.9–29.6]	
	277	17.9(50)	[13.0–24.1]	
Marital status				
Married/cohabiting	1049	13.4(141)	[10.1–17.4]	0.347
Widowed/divorced/separated	250	19.0(48)	[12.9–27.1]	
Single/never married	895	16.0(143)	[12.0–21.0]	
Socio-economic factors				
Education level				
No/primary school	871	19.6(171)	[15.2–25.0]	<0.05
Secondary school	823	12.8(105)	[9.2–17.7]	
Matric/higher	507	10.3(52)	[6.9–15.3]	
Employment status				
Unemployed	1346	15.8(213)	[12.7–19.5]	0.507
Employed	853	13.9(119)	[10.1–18.9]	
Household income				
R0-R2000	1138	15.0(171)	[11.7–19.0]	0.431
R2001-R5500	716	15.7(112)	[11.4–21.3]	
R5501 and more	149	8.2(12)	[2.7–22.2]	
Ever ran out of food				
Never	648	10.0(65)	[6.8–14.5]	<0.05
Sometimes	1271	16.3(207)	[13.1–20.1]	
Always	281	21.9(62)	[13.8–32.9]	
Living Standard Measure				
Low	723	14.3(103)	[10.2–19.7]	0.133
Medium	725	18.3(133)	[13.6–24.2]	
High	783	11.9(93)	[8.9–15.9]	
Health and behavioural factors				
Illness or injury				
No	1663	11.4(190)	[8.9–14.5]	<0.001
Yes	536	27.1(145)	[20.9–34.2]	
Smoke				
No	1397	14.1(197)	[11.3–17.6]	0.359
Yes	813	16.8(137)	[12.4–22.5]	
Alcohol use				
No	1441	15.2(219)	[12.2–18.8]	0.479
Yes	754	13.2(100)	[9.5–18.1]	
Drug use				
No	2046	15.3(313)	[12.7–18.2]	<0.001
Yes	109	1.4(2)	[0.5–3.8]	

CI = Confidence Interval. Subtotals are not always equal to the overall total due to non-response or missing data.

**Table 3:** Multivariate logistic regression model showing factors associated with deteriorated SPH status among informal settlement dwellers.

	Odds Ratio	[95%CI]	*p* Value
Demographic factors			
Sex			
Male (ref)			
Female	1.308	[0.755–2.265]	0.338
Age group			
18 to 29 (ref)			
30 to 39	0.332	[0.131–0.840]	<0.05
40 to 49	0.716	[0.231–2.218]	0.562
50 to 59	0.909	[0.307–2.695]	0.864
60+	1.158	[0.363–3.690]	0.804
Marital status			
Married/cohabiting (ref)			
Widowed/divorced/separated	0.794	[0.370–1.704]	0.553
Single/never married	1.116	[0.607–2.053]	0.725
Socio-economic factors			
Education level			
No/primary school (ref)			
Secondary school	0.682	[0.308–1.508]	0.344
Matric/higher	0.905	[0.415–1.974]	0.802
Employment status			
Unemployed (ref)			
Employed	1.739	[0.984–3.074]	0.057
Household income			
R0-R2000 (ref)			
R2 001-R5500	1.132	[0.609–2.105]	0.694
R5501 and more	0.365	[0.144–0.922]	<0.05
Ever ran out of food			
Never (ref)			
Sometimes	1.840	[0.979–3.458]	0.058
Always	3.120	[1.258–7.737]	<0.05
Living Standard Measure			
Low (ref)			
Medium	1.179	[0.593–2.342]	0.638
High	0.813	[0.369–1.793]	0.608
Health and behavioural factors			
Illness or injury			
No (ref)			
Yes	3.645	[2.147–6.186]	<0.001
Smoke			
No (ref)			
Yes	1.155	[0.605–2.203]	0.662
Alcohol use			
No (ref)			
Yes	1.172	[0.614–2.237]	0.629
Drug use			
No (ref)			
Yes	0.069	[0.020–0.240]	<0.001

CI = Confidence Interval.

**Table 4:** Multinomial logistic regression models showing factors associated with deteriorated SPH status among informal settlement dwellers.

	Model 1—Better as Base Category			Model 2—Neutral as Base Category		
	OR	[95%CI]	*p* Value	OR	[95%CI]	*p* Value
Sex						
Male (ref)						
Female	1.339	[0.766–2.341]	0.301	1.257	[0.772–2.047]	0.352
Age group						
18 to 29 (ref)						
30 to 39	0.367	[0.161–0.838]	0.018	0.29	[0.086–0.978]	0.046
40 to 49	0.832	[0.279–2.481]	0.737	0.595	[0.164–2.167]	0.426
50 to 59	1.107	[0.433–2.830]	0.83	0.725	[0.205–2.559]	0.612
60+	1.378	[0.513–3.699]	0.519	0.934	[0.308–2.837]	0.903
Marital status						
Married/cohabiting (ref)						
Widowed/divorced/separated	0.871	[0.434–1.748]	0.693	0.73	[0.408–1.306]	0.284
Single/never married	1.104	[0.847–1.439]	0.457	1.124	[0.650–1.945]	0.671
Socio-economic factors						
Education						
No/primary school (ref)						
Secondary school	0.726	[0.402–1.312]	0.284	0.638	[0.374–1.087]	0.097
Matric/higher	1.045	[0.356–3.069]	0.935	0.771	[0.217–2.737]	0.683
Employment						
Unemployed (ref)						
Employed	1.654	[0.881–3.106]	0.115	1.83	[1.001–3.347]	0.05
Household income						
R0-R2000 (ref)						
R2001-R5500	1.368	[0.592–3.159]	0.457	0.92	[0.455–1.862]	0.814
R5501 and more	0.446	[0.130–1.533]	0.196	0.296	[0.078–1.125]	0.073
Ever an out food						
Never (ref)						
Sometimes	1.899	[1.173–3.076]	0.01	1.785	[1.108–2.875]	0.018
Always	5.168	[1.563–17.090]	0.008	1.996	[0.982–4.055]	0.056
Living Standard Measure						
Low (ref)						
Medium	1.11	[0.628–1.963]	0.715	1.257	[0.620–2.550]	0.52
High	0.759	[0.273–2.110]	0.592	0.874	[0.324–2.357]	0.787
Health and behavioural factors						
Illness or injury						
No (ref)						
Yes	3.377	[2.137–5.335]	<0.001	3.979	[2.638–6.002]	<0.001
Smoke						
No (ref)						
Yes	1.215	[0.843–1.751]	0.29	1.083	[0.589–1.990]	0.796
Alcohol use						
No (ref)						
Yes	1.177	[0.545–2.542]	0.673	1.174	[0.588–2.343]	0.645
Drug use						
No (ref)						
Yes	0.058	[0.016–0.211]	<0.001	0.082	[0.023–0.289]	<0.001

CI = Confidence Interval. OR = Odds Ratio.

## Data Availability

Available on request from corresponding author at a reasonable time.

## Supplementary Materials

The following supporting information can be downloaded at: https://www.mdpi.com/article/10.3390/ijerph20054174/s1, File S1: Household Questionnaire 2015.

## Appendix B

**Table A1:** List of Assets.

Asset	Response	n
Hot running water	No	2240
	Yes	48
Fridge	No	1404
	Yes	900
Deep freezer	No	2095
	Yes	225
Domestic servant	No	2263
	Yes	27
VCR/DVD	No	1550
	Yes	622
Vacuum cleaner	No	2220
	Yes	90
Cell phone	No	669
	Yes	1662
Washing machine	No	2078
	Yes	243
Computer	No	2223
	Yes	95
Internet access	No	2205
	Yes	110
Electric/gas stove without oven	No	1440
	Yes	877
TV	No	1302
	Yes	1028
Tumble dryer	No	2263
	Yes	51
Telephone	No	2279
	Yes	28
Radio	No	1690
	Yes	632
HI/FI Music	No	2126
	Yes	184
Built in kitchen	No	2234
	Yes	73
Home security service	No	2294
	Yes	13
Microwave oven	No	1917
	Yes	404
M-NET/DSTV	No	2139
	Yes	177
Dishwashing machine	No	2298
	Yes	15
Sewing machine	No	2274
	Yes	37
Car	No	2132
	Yes	179
Iron	No	1153
	Yes	1169
Electric/gas stove with oven	No	1901
	Yes	411
Water tank	No	2278
	Yes	37
Power generator	No	2266
	Yes	47
Fan	No	2158
	Yes	151
Mattress	No	473
	Yes	1874
Bicycle	No	2146
	Yes	164
Motorcycle/scooter	No	2297
	Yes	13
Truck	No	2304
	Yes	6
Cart	No	2295
	Yes	11
Animals	No	2209
	Yes	95
Tools	No	1635
	Yes	649
