# Supplementary material for: Determinants of Deteriorated Self-Perceived Health Status among Informal Settlement Dwellers in South Africa
Source: Int J Environ Res Public Health. 2023 Feb 26;20(5):4174. doi: 10.3390/ijerph20054174 (PMC10001468; doi:10.3390/ijerph20054174)
Supplement: Supplementary file 1 [file ijerph-20-04174-s001.zip › ijerph-2139055-supplementary.pdf]

A BASELINE STUDY FOR FUTURE IMPACT EVALUATION FOR INFORMAL SETTLEMENTS TARGETED FOR UPGRADING

HOUSEHOLD QUESTIONNAIRE

LANGUAGE OF CHOICE TO BE USED.

GEOGRAPHIC PARTICULARS									
**QUESTIONNAIRE NUMBER:**	0	0	0	0					
**PROVINCE[1]:**	1 = WC	2 = EC	3 = NC	4 = FS	5 = KZN	6 = NW	7 = GP	8 = MP	9 = LP
**INFORMAL SETTLEMENT NAME:**									
**INFORMAL SETTLEMENT CODE** ***(Please enter the code of the informal settlement as per Informal Settlement Code Sheet)***									
**VISITING POINT (VP) NUMBER (FROM MAP):**									
**GPS CO-ORDINATES:** *(For mismatches)*	**GPS COORDINATES OF DWELLING: LONGITUDE** ○ .				**GPS COORDINATES: LATITUDE** ○ .				
**NUMBER OF HOUSEHOLDS AT VISITING POINT:**									
**SELECTED HOUSEHOLD TAG NUMBER:**	C	O	D	E					
**ADDRESS OF DWELLING: (IF APPLICABLE)**									

[1] 1 = Western Cape (WC) 2 = Eastern Cape (EC) 3 = Northern Cape (NC) 4 = Free State (FS) 5 =KwaZulu-Natal (KZN) 6 = North West (NW) 7 = Gauteng (GP) 8 = Mpumalanga (MP) 9 = Limpopo (LP)

PARTICULARS OF VISITS							
	Year	Month		Day		Time code	Response code
First visit	2015						
Second visit	2015						
Third visit	2015						
**Final response code**							

**Time code**	**Response code**	
1 = Morning till 12h00<br>2 = 12h01-15h00<br>3 = 15h01-18h00<br>4 = 18h01-21h00<br>5 = 21h01 and later	1 = Interview completed<br>2 = Interview partly completed and another appointment made<br>3 = Appointment made for interview<br>4 = Not a valid visiting point<br>5 = No one living here (unoccupied)	6 = No one at home<br>7 = No one at home for duration of the survey<br>8 = Refusal by household head<br>9 = No one eligible to complete questionnaire<br>10 = Incapacitated/Child-headed (specify) ___________________________<br>11 = Other (specify)

**Interview:**	**Name:**											
	**Staff number:**											

	H	H	M	M
**Interview starting time:**	H	H	M	M
**Interview finishing time:**	H	H	M	M

NB: HOUSEHOLD MEMBERSHIP CRITERIA

You are a household member if:

(i) You have lived under this "roof" or within the same compound/homestead/stand AT LEAST 15 DAYS DURING THE LAST 12 MONTHS OR YOU ARRIVED HERE IN THE LAST 15 DAYS and this is NOW YOUR USUAL RESIDENCE and

(ii) when you are/were together you SHARE/D FOOD FROM A COMMON SOURCE with other household members and

(iii) when you are/were together you CONTRIBUTE/D TO OR SHARE/D IN A COMMON RESOURCE POOL.

FIELDWORKER: OBSERVE AND COMPLETE

A. The type of dwelling that the household occupies.

INSTRUCTION TO INTERVIEWER*: Ask the respondent which one is the MAIN dwelling. Please circle ONE option*

Dwelling/house **OR** brick/concrete block structure on a separate stand or yard or on farm	1
Traditional dwelling/hut/structure made of traditional materials (Wattle & Daub[1]/mud)	2
Double-storey dwelling	3
Dwelling/house/flat/room in backyard	4
Shack (plastic/semi-permanent material/corrugated iron/cardboard)	5
Shipping Containers	6
Caravan/Tent	7
Other, Specify	8

[1]Wattle & Daub is a traditional construction method that is used in rural areas. The house wall consists of wooden sticks or poles that are covered with mud or a mixture of mud and clay.

The materials used for the WALLS and ROOF of the MAIN dwelling.
INSTRUCTION TO INTERVIEWER*: Multiple responses allowed.*

	**B. Walls**	**C. Roof**
Bricks	1	1
Cement block/concrete	2	2
Corrugated iron/zinc	3	3
Wood/Timber	4	4
Plastic/Marquee material/Cloth	5	5
Cardboard	6	6
Mud and cement mix	7	7
Wattle and daub	8	8
Tile	9	9
Mud/Clay	10	10
Thatching/grass	11	11
Asbestos	12	12
Other (specify) ►	13	13

D. The materials of the FLOORS of the dwelling.

Tile/ceramic	1
Concrete	2
Plastic/Marquee material/Cloth	3
Mud/clay	4
Wood/Timber	5
Other (specify) ►	6

In what condition are the walls and the roof of the main dwelling?

	**E. Walls**	**F. Roof**
Very Weak *(More than ten cracks/defects on wall/roof)*	1	1
Weak *(Three and Nine cracks/defects on wall/roof)*	2	2
Need minor repairs *(One or two cracks/defects on wall/roof)*	3	3
Good *(Superficial cracks/defects on wall/roof)*	4	4
Very good *(No cracks/defects on wall/roof)*	5	5

BASELINE STUDY FOLLOW-UP CONTACT DETAILS

INTERVIEWER READ OUT: We contact some respondents to make sure you are happy with the way this interview was undertaken and to confirm we have taken your answers correctly. We would like to confirm your contact details. If respondent is not head of household please ask for the details of the head. If respondent does not know or refuses to give details of head ask for their details.

**G. ID of Head of Household**:													

H. Please indicate whose details are being captured: The following details are for (please tick one option): ☐ 1 = Head of Household; ☐ 2 = Respondent Not Head of Household

**I.**	What would be the best number to contact you on DURING THE DAY?<br>***Interviewer:*** *Include area codes for landline numbers*	NAME: ▼	TEL. NUMBER: ►
**J.**	What would be the best number to contact you on DURING THE EVENING?<br>***Interviewer:*** *Include area codes for landline numbers*	NAME: ▼	TEL. NUMBER: ►
**K.**	Is there any other number we ought to try if we cannot get you on the number/s you have given us?<br>***Interviewer:*** *Include area codes for landline numbers*	NAME: ▼	TEL. NUMBER: ►

MODULE 1: HOUSEHOLD ROSTER

A household is a group of persons related or not, living under the same roof, **under the responsibility of a head whose authority is acknowledged by all the members**. The ordinary household is composed of a head of household, his spouse(s), his unmarried children, and **possibly** his relatives or other persons **to whom he is unrelated**.

NB: Enter responses for household members in the same order as on the IDENTIFICATION FLAP.

PERSON CODE	**1.1. First name**<br>Write down first name of each member of the household, start the list exactly **AS YOU ENTERED IT ON THE IDENTIFICATION FLAP** with the **head of the household** then from oldest to youngest.	**1.2. Does [NAME] usually reside here at least 4 nights a week?**<br>1 = Yes<br>2 = No	**1.3. Relationship to Head of the Household**<br>1 = Head/acting head of the household<br>2 = Wife or husband or partner of **01**<br>3 = Son/daughter/stepchild/adopted child **01**<br>4 = Father/mother/step mother/step father<br>5 = Brother/sister/step brother/step sister<br>6 = Grandchild/great grandchild<br>7 = Grandparent/great grandparent<br>8 = Mother- or father-in-law<br>9 = Son- or daughter-in-law<br>10 = Brother- or sister-in-law<br>11 = Other relative (specify)<br>12 = No-relation (specify)	**1.4. Indicate respondent who answers the question**	**1.5. Gender?**<br>1 = Male<br>2 = Female	**1.6. What year was [NAME] born?**<br>If Respondent Does Not Know Day, Month Write '99'. For Year Write '9999'.<br>**DD/MM/YYYY**<br>**E.G. 18/05/2015**			**1.7. What is [NAME'S] race?**<br>1 = Black African<br>2 = Coloured<br>3 = Asian/Indian<br>4 = White<br>5 = Other	**1.8. Which ONE of the following languages do you speak the most at home?**<br>1 = Afrikaans<br>2 = English<br>3 = IsiNdebele<br>4 = Sepedi<br>5 = Sesotho<br>6 = Seswati<br>7 = Setswana<br>8 = Tshivenda<br>9 = IsiXhosa<br>10 = IsiZulu<br>11 = Xitsonga<br>12 = Other
						**DD**	**MTH**	**YEAR**		
1	HEAD OF HOUSEHOLD									
2										
3										
4										
5										
6										
7										
8										
9										
10										
11										
12										
13										
14										
15										
16										
17										
18										

INTERVIEWER: *Household members take the same person code as assigned on the identification flap.*

PERSON CODE	**1.9. How many children does [NAME] have? Write 0 if person doesn't have any children.**<br>This includes children not residing with the household member. For couples/married individuals staying together please enter number of children for one parent.	**1.10. FOR FEMALES older than 15 years: Is [NAME] expecting a child?**<br>1 = Yes<br>2 = No<br>3 = Refused to answer<br>8 = Don't know	**1.11. Was [NAME] born in this settlement?**<br>1 = Yes<br>2 = No<br>8 = Don't know	**1.12. How long has [NAME] been living in this settlement?**<br>If 1 month or less, write **0000** for years, and **01** for month.<br>Enter year in full CCYY e.g. **2014**<br>Enter **9999** for year and **99** for month if respondent does not know.		**1.13. How many months has [NAME] been away from the household in the last 12 months?**<br>If less than 1 MONTH, write **01** for MONTH.<br>Enter **99** for month if respondent does not remember.		**1.14. What is the [NAME] present marital status?**<br>1 = Married (Civil marriage)<br>2 = Married (Custom/traditional marriage-lobola)<br>3= Living together (not married)<br>4 = Widow/Widower<br>5 = Divorced or Separated<br>6 = Single/Never Married	**1.15. Does [NAME] have a bar coded ID (green ID with a bar code in it)?**<br>1 = Yes<br>2 = No<br>8 = Don't Know/No Response
				**Years**	**Mths**		**Mths**		
1	HEAD OF HOUSEHOLD								
2									
3									
4									
5									
6									
7									
8									
9									
10									
11									
12									
13									
14									
15									
16									
17									
18									

INTERVIEWER: *Household members take the same person code as assigned on the identification flap.*

PERSON CODE	**1.16. What is [NAME] nationality?**<br><br>1 = South African **(Skip Q1.17 & Q1.18)**<br>2 = Other African<br>3 = European<br>4 = Asian<br>5 = Other<br>8 = Do not know/no answer	**1.17. If not South African, what is [NAME]'s legal status?**<br><br>1 = Permanent resident<br>2 = Temporary resident<br>3 = Refugee<br>4 = Does not have proper documents<br>5 = Other<br>8 = Do not know<br>9 = Refused to Answer	**1.18. If refugee does [NAME] have a refugee ID card?**<br><br>1 = Yes<br>2 = No<br>8 = Don't know/No response	**1.19. Do any of the household members visit rural areas?**<br><br>1 = Yes<br>2 = No	**1.20. How often does [NAME] visit rural area?**<br><br>1 = Once a month<br>2 = Once every 3-6 months<br>3 = Once every 12 months<br>4 = No visits/ No rural house
1	HEAD OF HOUSEHOLD				
2					
3					
4					
5					
6					
7					
8					
9					
10					
11					
12					
13					
14					
15					
16					
17					
18					

MODULE 2: EDUCATION *Household members take the same person code as assigned on the identification flap.*

PERSON CODE	**2.1. Can [NAME] read and write?**<br><br>1 = Cannot read and write<br>2 = Read Only<br>3 = Read and Write	**2.2. What is the HIGHEST level of education that [NAME] has successfully completed?**<br>00 = No Schooling<br>01 = Grade R/0<br>02 = Grade 1/ Sub A<br>03 = Grade 2 / Sub B<br>04 = Grade 3/Standard 1<br>05 = Grade 4/ Standard 2<br>06 = Grade 5/ Standard 3<br>07 = Grade 6/Standard 4<br>08 = Grade 7/Standard 5<br>09 = Grade 8/Standard 6/Form 1<br>10 = Grade 9/Standard 7/Form 2<br>11 = Grade 10/ Standard 8/ Form 3<br>12 = Grade 11/ Standard 9/ Form 4<br>13 = Grade 12/Standard 10/Form 5/Matric (NQF 4)<br>14 = Higher certificate (NQF 5)<br>15 = Diploma / Advanced certificate (NQF 6)<br>16 = Bachelor's degree / Advanced diploma (NQF7)<br>17 = Honours degree / Post grad diploma (NQF 8)<br>18 = Higher Degree (MA/PHD [NQF 9/10])<br>19 = Don't Know<br>20 = Other (Specify)	**2.3. Is [NAME] currently enrolled in crèche or primary school or high school?**<br><br>1 = Yes **(If YES skip to Q2.5)**<br>2 = No<br>3 = Not Applicable	**2.4. What is the main reason why [NAME] stopped attending / did not attend school?**<br>1 = Not Keen To Attend<br>2 = Could Not Get Into School<br>3 = To Help With Housework<br>4 = Suspended<br>5 = No money for Fees, Books, and Materials<br>6 = Long Distance To School<br>7 = Dangerous Route To School<br>8 = Cost Of Transport<br>9 = Sick<br>10 = Graduated<br>11 = Not Of School-Going Age<br>12 = He or she is working at home or business/job<br>13 = Not Applicable<br>14 = Other (Specify)<br>________________	**2.5. Does [NAME] attend crèche or primary school or high school [OPTION]?**<br><br>1 = In this settlement **(Go to Q3.1)**<br>2 = In a neighbouring settlement<br>3 = In town<br>4 = Nearby town<br>5 = Other (Specify)<br>________________	**2.6. If not in this settlement, why does [NAME] not go to school in this settlement?**<br><br>1 = No school available<br>2 = Poor school quality<br>3 = Too expensive<br>4 = Too far away<br>5 = No public transport links<br>6 = Friends are at another school<br>7 = Other (Specify)<br>________________
1						
2						
3						
4						
5						
6						
7						
8						
9						
10						
11						
12						
13						
14						
15						
16						
17						
18						

MODULE 3: ECONOMIC ACTIVITY *Household members take the same person code as assigned on the identification flap.*

***INTERVIEWER*: ASK FOR ALL HOUSEHOLD MEMBERS 15 YEARS AND OLDER. (Answer 3.1(a)-(c) and then CAREFULLY follow the skip instructions)**

PERSON CODE	**3.1. (a) During the last calendar week (Sunday to Saturday) did [NAME] work for a wage, salary, commission or any payment in kind (including paid domestic work), even if it was for only one hour?**<br>*Examples:* a regular job, contract, casual or piece work for pay, work in exchange for food or housing, paid domestic work.<br><br>1 = Yes<br>2 = No<br>3 = Don't Know	**3.1 (b) During the last calendar week (Sunday to Saturday) did [NAME] run or do any kind of business, big or small, for yourself or with one or more partners, even if it was for only one hour?** *Examples*: Commercial farming, selling things, making things for sale, construction, repairing things, guarding cars, brewing beer, collecting wood or water for sale, hairdressing, crèche businesses, taxi or other transport business, having a legal or medical practice, performing in public, having a public phone shop, etc.<br>1 = Yes<br>2 = No<br>3 = Don't Know	**3.1 (c) During the last calendar week (Sunday to Saturday) did [NAME] help without being paid in any kind of business, run by his/her household even if it was for only one hour**? *Examples*: Commercial farming, production of agricultural produce to sell, help to sell things, make things for sale or exchange, doing the accounts, cleaning up for the business, etc.<br><br>1 = Yes<br>2 = No<br>3 = Don't Know	**NB: If YES to ANY of Q3.1 (a) Q3.1 (b) Q3.1 (c) Ask Q3.2; Q3.3 and Q3.4**<br><br>**If response is 2 = No OR 3 = Don't Know to ALL of Q3.1 (a) Q3.1 (b) Q3.1 (c) Then Go to Q3.5 for that household member.**	**3.2. How many hours did [NAME] work last week?**<br>NB. A typical work day is 8 hrs. A 5 day work week, Mon-Fri has 40 hours. Probe to find out how many hours worked based on days.<br>**ENTER HOURS BASED ON RESPONSE**<br><br>**Enter the following codes for...**<br>Refused = 996<br>Don't Know = 997<br>Not Applicable = 999	**3.3. Would [NAME] have liked to work more hours than they actually worked last week, provided the extra hours had been paid?**<br><br>1 = YES, in the current job<br>2 = YES, in taking an additional job<br>3 = YES, in another job with more hours<br>4 = No<br>8 = Don't Know
1	HEAD OF HOUSEHOLD					
2						
3						
4						
5						
6						
7						
8						
9						
10						
11						
12						
13						
14						
15						
16						
17						
18						

P E R S O N C O D E	**3.4. Is the organization/business/branch where [NAME] works**<br>1 = In the FORMAL SECTOR (registered to perform activity) **(Go to Q3.10)**<br>2 = In the INFORMAL SECTOR (not registered to perform activity) **(Go to Q3.10)**<br>3 = Do not know **(Go to Q3.10)**	**3.5. In the last calendar week (Sunday to Saturday), even though [NAME] did not do any work for pay or profit, does [NAME] have a job or business that he/she would definitely return to?**<br><br>1 = Yes **(Go to Q3.10)**<br>2 = No<br>3 = Don't Know	**3.6. During the last four calendar weeks, was [NAME] looking for any kind of job or trying to start any type of business?**<br><br>1 = Yes **(Go to Q3.9)**<br>2 = No<br>3 = Don't Know	**3.7. Would [NAME] have liked to work during the last calendar week (Sunday to Saturday)?**<br><br>1 = Yes<br>2 = No **(Go to Q3.10)**<br>3 = Don't Know **(Go to Q3.10)**	**3.8. What was the main reason for not trying to find work or starting a business during the past four calendar weeks?**<br>1 = Awaiting the season for work<br>2 = Waiting to be recalled to former job<br>3 = Health reasons<br>4 = Pregnancy<br>5 = Disabled or Unable to work (Handicapped)<br>6 = Housewife/Homemaker (Family considerations/child care)<br>7 = Undergoing training to help find work<br>8 = No jobs available in the area<br>9 = Lack of money to pay for transport to look for work<br>10 = Unable to find work requiring his/her skills<br>11 = Lost hope of finding any kind of work<br>12 = No transport available<br>13 = Scholar or student<br>14 = Retired<br>15 = Too old/young to work<br>16 = Did not want to work<br>17 = Other	**3.9. If a suitable job had been offered or circumstances had allowed, would [NAME] have been able to start work or a business in the last calendar week (Sunday to Saturday)?**<br><br>1 = Yes<br>2 = No<br>3 = Don't Know	**3.10.** ***ASK FOR ALL HOUSEHOLD MEMBERS ABOVE 15:*** **Has …… participated in a Government or municipal job creation programme or expanded public works programme in the past 6 months? This includes community based workers such as community development workers, home based care workers etc.**<br><br>1 = Yes<br>2 = No<br>3 = Don't Know
1							
2							
3							
4							
5							
6							
7							
8							
9							
10							
11							
12							
13							
14							
15							
16							
17							
18							

3.11. ***ASK ALL RESPONDENTS:*** **Compared to TWO years ago, has the unemployment levels in your community changed or remained the same? (Circle one)**

Much Worse	1	Somewhat Better	4
Somewhat Worse	2	Much Better	5
About The Same	3		

3.12. Did [NAME] earn any income from [OPTIONS BELOW] in the last month? Ask respondent each option, if YES enter amount if NO Move to next option.
YES ► How much did [NAME] earn from [OPTIONS BELOW] **last month** (FILL IN BELOW, ROUNDED TO THE NEAREST RAND)?

PERSON CODE	OPTIONS									
	**Income from employment (wages/ salary) / income business**	**Family transfer**	**Help from friends**	**Pension (not from government grant)**	**Old Age Pension Less than 75 years = R1,428 Older than 75 years = R1,449**	**Child support (own child) (R330)**	**Foster child support (R830)**	**Disability (R1,410)**	**Care dependency (disabled child) (R1,410)**	**Other (specify)**
1	R	R	R	R	R	R	R	R	R	R
2	R	R	R	R	R	R	R	R	R	R
3	R	R	R	R	R	R	R	R	R	R
4	R	R	R	R	R	R	R	R	R	R
5	R	R	R	R	R	R	R	R	R	R
6	R	R	R	R	R	R	R	R	R	R
7	R	R	R	R	R	R	R	R	R	R
8	R	R	R	R	R	R	R	R	R	R
9	R	R	R	R	R	R	R	R	R	R
10	R	R	R	R	R	R	R	R	R	R
11	R	R	R	R	R	R	R	R	R	R
12	R	R	R	R	R	R	R	R	R	R
13	R	R	R	R	R	R	R	R	R	R
14	R	R	R	R	R	R	R	R	R	R
15	R	R	R	R	R	R	R	R	R	R
16	R	R	R	R	R	R	R	R	R	R
17	R	R	R	R	R	R	R	R	R	R
18	R	R	R	R	R	R	R	R	R	R

3.13. What are the sources of income for this household?
Please consider the income of all household members and any income which may be received by the household as a whole.
Read all the options

	1 = Yes	2 = No
1. Salaries/wages/commission	1	2
2. Income from a business	1	2
3. Remittances (money received from people living elsewhere)	1	2
4. Pensions (exclude old age state grant)	1	2
5. Grants (include old age grant here)	1	2
6. Sales of farming products and services	1	2
7. Other income sources e.g. rental income, interest	1	2
8. No income	1	2
9. Informal trading	1	2

3.14. Which one of the ABOVE income sources is the main source of income?
Enter option number (e.g. if income from a business enter 2) ☐

3.15. Please indicate the INCOME BAND that best describes the COMBINED TOTAL MONTHLY HOUSEHOLD INCOME (3.15a) of all the people in your household before tax and other deductions. This TOTAL should include all sources of income, i.e. salaries, pensions, income from investment, etc.
If household income varies seasonally ask for TOTAL ANNUAL HOUSEHOLD INCOME (3.15b).

**3.15a MONTHLY Household Income**	
No income	1
R1 - R500	2
R501 - R1,000	3
R1,001 - R1,500	4
R1,501 - R2,000	5
R2,001 - R2,500	6
R2,501 - R3,000	7
R3,001 - R3,500	8
R3,501 - R4,500	9
R4,501 - R5,500	10
R5,501 - R6,500	11
R6,501 - R7,500	12
R7,501 - R8,500	13
R8,501 - R9,500	14
R9,501 - R10,500	15
R10,501 - R12,500	16
R12,501 - R14,500	17
R14,501 - R16,500	18
R16,501 - R18,500	19
R18,501 - R20,500	20
R20,501 - R30,500	21
R30,501 - OR MORE	22
Refuse to answer	23
Not sure	24

**3.15b ANNUAL Household Income**	
No income	1
R1 - R6,000	2
R6,001 - R12,000	3
R12,001 - R18,000	4
R18,001 - R24,000	5
R24,001 - R30,000	6
R30,001 - R36,000	7
R36,001 - R42,000	8
R42,001 - R54,000	9
R54,001 - R66,000	10
R66,001 - R78,000	11
R78,001 - R90,000	12
R90,001 - R102,000	13
R102,001 - R114,000	14
R114,001 - R126,000	15
R126,001 - R150,000	16
R150,001 - R174,000	17
R174,001 - R198,000	18
R198,001 - R222,000	19
R222,001 - R246,000	20
R246,001 - R366,000	21
R366,001 - OR MORE	22
Refuse to answer	23
Not sure	24

**3.16. In total, how much did the household spend on the following categories last month?**		**LIVING STANDARDS MEASURE**<br>**3.17. Please tell me which of the following, if any, are presently in your household (IN WORKING ORDER)? Do you have …**<br>1 = YES<br>2 = NO<br>If household does not know when acquired enter **9999** as response code.<br>**Ask the respondent the year when the asset was acquired. Enter the year in full CCYY e.g. 2014**					
**CATEGORY**	**RAND**		1 = Yes<br>2 = No	**Year Acquired**		1 = Yes<br>2 = No	**Year Acquired**
Food	R	Hot running water			Microwave oven (in working order)		
Transport	R	Fridge			M-Net and or DStv subscription		
Education	R	A deep freezer (in working order)			A dishwashing machine		
Health	R	Full time domestic servant			A sewing machine		
Housing rent, if any	R	VCR/DVD in household			A motor vehicle/Car		
Household services (water, electricity)	R	Vacuum cleaner/floor polisher			An iron (electric or coal)		
Household levies/rates and taxes	R	Any Cell phone in household			Electric /gas stove with an oven		
Debts	R	A washing machine			Water Tank		
Transfers to other households	R	A computer at home			Power generator		
Entertainment	R	Internet access			Fan		
Cigarettes/Tobacco/Alcohol	R	An electric / gas stove without oven			Mattress		
Business	R	A TV set			Bicycle		
Cell phone airtime	R	A tumble dryer			Motorcycle/Scooter		
Baby products	R	A Telkom home telephone			Truck		
Clothes	R	A Radio			Cart		
School fees	R	Hi-fi/music centre			Pack animals (donkey. horse)		
Other (Specify) ▶	R	Built in kitchen sink			Tools		
Other (Specify) ▶	R	Home security service					

3.18. What is the gross total household income per month? (Ask respondent to include all income sources) R

3.19. How much did your household spend on energy in the last month? This would include expenses on all (electricity and other fuels)

Amount (In Rands)		(Don't Know)	(Refused)
**R**		**8**	**9**

3.20. On average, how much does your household spend each month on the following energy sources?

	Energy cost (Rands)
1. Paraffin	R
2. Gas	R
3. Candle	R
4. Coal	R
5. Firewood	R
6. Solar system	R
7. Electricity	R
8. Batteries	R
9. Car batteries	R
10. Generator (petrol/diesel)	R
11. Other (specify) ►	R

3.21. Do you consider your household to be poor? ☐ 1 = Yes ☐ 2 = No

Please imagine a six (6) step ladder where the poorest people in South Africa stand on the bottom (the first step) and the richest people in South Africa stand on the highest step (the sixth step).

	Poorest					Richest
**3.22. On which step was your household 3 years ago?**	1	2	3	4	5	6
Don't Know	8					
Refused	9					

**3.23. On which step are you today?**	1	2	3	4	5	6
Don't Know	8					
Refused	9					

3.24. How would you compare your household standard of living in relation to other households in this community? (Circle one)

Much Worse	1
Somewhat Worse	2
About the Same	3
Somewhat Better	4
Much Better	5

3.25. Are you happier, the same or less happy with life than you were 5 years ago?

Happier	1
The same	2
Less happy	3
Refused	8
Don't Know	9

MODULE 4: HEALTH, FOOD AND NUTRITION SECURITY

**While in this settlement…**	1 = Yes	2 = No
**4.1. …has this household experienced the death of a child before their first birthday?**	1	2
**4.2. …and in the LAST 12 MONTHS has this household experienced the death of a child under the age of 5 years?**	1	2
**4.3. … have any of the females in this household experienced a miscarriage, still birth of abortion?**	1	2
**4.4. … and in the last 12 months has anyone in your household had tuberculosis?**	1	2

4.5. Tell us about the food and nutrition situation in your household. During the past 12 months, was there a time when:

	1 = Never	2 = Sometimes	3 = Always
1. The family was worried that it would run out of food?	1	2	3
2. The family was unable to eat healthy and nutritious* food?	1	2	3
3. The family ate only a few kinds of foods?	1	2	3
4. Some family members had to skip a meal?	1	2	3
5. The family ate less than it thought it should?	1	2	3
6. Your household ran out of food?	1	2	3
7. Someone in the family was hungry but did not eat?	1	2	3
8. Someone in the family went without eating for a whole day?	1	2	3
**FOR HOUSEHOLDS WITH CHILDREN < 5 years of age**	1	2	3
**4.6. During the last 12 MONTHS, was there a time when any of the children younger than 5 years old:**	1	2	3
9. Did not eat healthy and nutritious foods because of a lack of money or other resources?	1	2	3
10. Was not given enough food because of a lack of money or other resources?	1	2	3

*Nutritious food consists of all six categories of nutrients that the body needs, that is, protein, carbohydrates, fat, fibres, vitamins and minerals, and water. It should include eatables like vegetables, fruits, meat, dairy, legumes (e.g. peas, beans) etc.

INTERVIEWER: *Household members take the same person code as assigned on the identification flap.*

PERSON CODE	**4.7. Has [NAME] suffered from an illness or injury during the past 4 weeks?**<br>1 = Yes<br>2 = No **(Skip to Q4.11)**	**4.8. What was the nature of the main illness, injury, disability or ailment that [name] suffered from in the past 4 weeks?**<br>1 = Flu<br>2 = Fever<br>3 = Diarrhoea<br>4 = Cold<br>5 = Worms<br>6 = High Blood Pressure<br>7 = Measles<br>8 = Hepatitis B<br>9 = Stomach Pain<br>10 = Skin Irritation/Itching<br>11 = Allergies<br>12 = Tuberculosis<br>13 = Asthma<br>14 = Malaria<br>15 = Injury<br>16 = Violence-Related Injury<br>17 = Illness Related To Pregnancy<br>18 = Cancer<br>19 = Diabetes<br>20 = HIV Infection<br>21 = Physical Disability (Specify)<br>22 = Mental Disability (Specify)<br>23 = Other (Specify)	**4.9. How many days in the past 4 weeks has [NAME] been sick or injured?**<br>ENTER NUMBER OF DAYS	**4.10. How many days in the past 4 weeks has [NAME] not been able to do what he/she normally does because of illness or injury?**<br>ENTER NUMBER OF DAYS	**4.11. Does [NAME] smoke tobacco e.g. cigarettes, cigars…?**<br>1 = Often<br>2 = Sometimes<br>3 = Never<br>4 = I Don't Know	**4.12. Does [NAME] consume alcohol?**<br>1 = Often<br>2 = Sometimes<br>3 = Never	**4.13. Does [NAME] abuse any substances (i.e. drugs)?**<br>1 = Often<br>2 = Sometimes<br>3 = Never<br>4 = I Don't Know
1							
2							
3							
4							
5							
6							
7							
8							
9							
10							
11							
12							
13							
14							
15							
16							
17							
18							

INTERVIEWER: *Household members take the same person code as assigned on the identification flap.*

PERSON CODE	**4.14. Compared with [NAME]'s health one year ago, how is his/her health now?**<br>**IF CHILD WAS BORN LESS THAN ONE YEAR AGO, ENTER '00'**<br>1 = Much Better<br>2 = Somewhat Better<br>3 = About the Same<br>4 = Somewhat Worse<br>5 = Much Worse	**4.15. Did any household member experience diarrhoea in the last month?**<br>1 = Yes<br>2 = No **(Go to Q4.19)**	**4.16. How many days was [NAME] sick with diarrhoea during this period?**	**4.17. Did [NAME] ever experience the following in his/her stool:**<br>1 = Blood<br>2 = Mucous<br>3 = Both<br>4 = Neither	**4.18. Was there any vomiting?**<br>1 = Yes<br>2 = No	**4.19. Did any household member experience breathing problems and/or chest infections in the last month?**<br>1 = Yes<br>2 = No **(Go to Q5.1)**	**4.20. How many days was [NAME] sick with a respiratory infection during this period?**	**4.21. Did [NAME] experience any cough?**<br>1 = Yes<br>2 = No	**4.22. Did [NAME] experience any breathing with sound?**<br>1 = Yes<br>2 = No	**4.23. Did [NAME] experience any rapid breathing?**<br>1 = Yes<br>2 = No
1										
2										
3										
4										
5										
6										
7										
8										
9										
10										
11										
12										
13										
14										
15										
16										
17										
18										

MODULE 5: PART A – BORROWING AND CREDIT

Please tell us about the credit practices of your household by answering the following questions.
INTERVIEWER: *Household members take the same person code as assigned on the identification flap.*

**PERSON CODE**	**5.1. Has any member of the household contracted any loan(s) [in cash and/or kind] or bought anything on credit over the past 12 months?**<br><br>1 = YES ► Continue<br>2 = NO ► **(Go to Q5.8)**	**5.2. Which household member obtained the loan?**<br><br>ENTER PERSON CODE	**5.3. What is the source of this loan/credit?**<br>1 = Bank<br>2 = Credit Card Company<br>3 = Government Agency<br>4 = NGO<br>5 = Business Firm<br>6 = Employer<br>7 = Money Lender<br>8 = Relative/ Neighbour/ Friend<br>9 = Community Centre<br>10 = Clothing/ Furniture/ Appliance Store<br>11 = Other (Specify)	**5.4. What was the total amount of the original loan/credit (give value if in kind)?**<br><br>ENTER RAND VALUE	**5.5. For what purpose was the loan contracted/credit used?**<br>1 = Investment in Property<br>2 = Agricultural Land/Equipment<br>3 = Agricultural Inputs<br>4 = Business<br>5 = Housing Upgrade<br>6 = Education/Training<br>7 = Health<br>8 = Ceremonies (weddings, funerals, etc.)<br>9 = Vehicle<br>10 = Clothing/ Furniture/ Appliances<br>11 = Other consumer goods<br>12 = Other (Specify)
1				R	
2				R	
3				R	
4				R	
5				R	
6				R	
7				R	
8				R	
9				R	
10				R	
11				R	
12				R	
13				R	
14				R	
15				R	
16				R	
17				R	
18				R	

PERSON CODE	**5.6. What kind of guarantee was required by the lender/credit provider?**<br>1 = None<br>2 = Land<br>3 = Cattle<br>4 = Furniture/TV<br>5 = House/Building<br>6 = Employment<br>7 = Relatives<br>8 = Non-Relatives<br>9 = ID / passport<br>10 = Other (Specify)	**5.7. Did you have the option to use your land or house / building as collateral/guarantee for this loan?**<br>1 = Yes<br>2 = No
1		
2		
3		
4		
5		
6		
7		
8		
9		
10		
11		
12		
13		
14		
15		
16		
17		
18		

5.8. Did this family ever run out of money to meet its day-to-day expenses in the last 12 months?

1 = Yes

2 = No **(If NO go to Q5.11)**

5.9. If YES which of the following coping strategies did the family adopt to deal with the situation? (MULTIPLE RESPONSE)

	1 = Yes	2 = No
1 = Borrowed money from the social group e.g. stokvel	1	2
2 = Borrowed money from a neighbour/relative	1	2
3 = Borrowed money from the bank/loan	1	2
4 = Borrowed money from an informal money lender	1	2
5 = Beg for basics like food	1	2
6 = Had to resort to stealing	1	2
7 = Looked for an extra job	1	2
8 = Sent the children away to reduce the cost of living	1	2
9 = Other, please specify_________________________________	1	2

5.10. If YES to OPTIONS 1/2/3/4, ABOVE, was the money paid back?

Yes	1
No	2
I Don't Know	9

MODULE 5: PART B – SAVINGS

INTERVIEWER: *Household members take the same person code as assigned on the identification flap.*

PERSON CODE	***5.11.*** **Does any member have a savings account or participate in any informal/semi-formal savings institution?** *Ask if the account is joint. If joint, capture for oldest member only.*<br>*We are only interested in savings for future spending.*<br>1 = Yes ▶ Fill up Table<br>2 = No ▶ Proceed to **MODULE 6**	**5.12. Which household member(s) is it?**<br>*If joint, capture for oldest member only.*<br>ENTER PERSON CODE	**5.13. What type of savings institution is it?**<br>1 = Rotating Savings & Credit Association / Stokvel<br>2 = Bank<br>3 = Cooperatives<br>4 = Credit Union<br>5 = Savings And Loan Group<br>6 = Other (Specify)<br>*If joint, capture for oldest member only.*	**5.14. What is the current balance of these savings?**<br>1 = R0<br>2 = R1 – R100<br>3 = R101 – R1,000<br>4 = R1001 – R2,000<br>5 = R,2001 - R4,000<br>6 = R4,001 or more	**5.15. Do you or any other member in the household plan to use the savings for [OPTIONS BELOW] in the next 12 months?**<br>***Ask for each option starting with (a).***					
					**a) Buying property (land or house)**		**b) Housing improvement**		**c) General house/plot maintenance**	
					1 = Yes	2 = No	1 = Yes	2 = No	1 = Yes	2 = No
1					1	2	1	2	1	2
2					1	2	1	2	1	2
3					1	2	1	2	1	2
4					1	2	1	2	1	2
5					1	2	1	2	1	2
6					1	2	1	2	1	2
7					1	2	1	2	1	2
8					1	2	1	2	1	2
9					1	2	1	2	1	2
10					1	2	1	2	1	2
11					1	2	1	2	1	2
12					1	2	1	2	1	2
13					1	2	1	2	1	2
14					1	2	1	2	1	2
15					1	2	1	2	1	2
16					1	2	1	2	1	2
17					1	2	1	2	1	2
18					1	2	1	2	1	2

MODULE 6: MICROENTERPRISE

**6.1. Over the past 12 months, has anyone in your household operated any NON-AGRICULTURAL ENTERPRISE which produces goods or services (for example, artisan, metalworking, tailoring, repair work; also include processing and selling your outputs from your own crops if done regularly) or has anyone in your household owned a shop or operated a trading business?**	Yes	1	► Continue
	No	2	► Proceed to MODULE 7

ENTERPISE ID	**6.2. What kind of enterprise does your household operate?**<br><br>1 = Spaza Shop<br>2 = Hair Salon<br>3 = Shebeen<br>4 = Tailor<br>5 = Car Or Electronics Repair<br>6 = Weaving<br>7 = Telephone Use<br>8 = Hawking/Selling Goods<br>9 = Other (Specify)	**6.3. Who in the household is most informed about and/or in charge of day-to-day operations of the enterprise?**	**6.4.** Where Possible, Have The Following Questions Answered By The Person Indicated In 6.3. IF THIS PERSON IS UNAVAILABLE, INDICATE THIS HERE. **Is the person indicated in Q6.3 available to answer questions Q6.5- Q6.10?**<br>1 = Yes<br>2 = No	**6.5. For how long has the enterprise been in operation (months and years)?**<br><br>If Respondent Does Not Know Month Write **'99'**.<br>If Respondent Does Not Know Year Write **'9999'**.		**6.6. Do you operate this business from home?**<br><br>1 = Yes<br>2 = No **(Go to Q6.8)**	**6.7. How many rooms in your home do you use for this business?**	**6.8. During the past 4 weeks, how many people did this enterprise employ?**<br><br>(MORE THAN 4 HOURS A DAY ON AVERAGE. EXCLUDE THE OPERATOR OF THE ENTERPRISE)	**6.9. What is the amount of total sales in a 'usual' sales month (four weeks)?**	**6.10. In a 'usual' sales month, how much have you spent in total on the purchase of inputs (labour, raw materials, items for resale, transport, electricity, water, fuel, rental, maintenance, taxes, registration fees, insurance, etc.)? (TOTAL COSTS)**
	**Write description (if other)**	**ID CODE**	**CODE**	**MTH**	**YEAR**		**NUMBER**	**NUMBER**	**RANDS**	**RANDS**
1									R	R
2									R	R
3									R	R
4									R	R
5									R	R
6									R	R

MODULE 7: HOUSING AND TENURE

I am going to ask you a few questions relating to your current accommodation.

7.1. When did you start living in this unit? (write the year below e.g. 2004)

7.2. Were you the first person/people to live in this dwelling?

Yes	1
No	2
Do not know	99

7.3. How did you come to live in this settlement? (List the 3 main reasons in order of importance, with 1 being the most important)

Forced to relocate		1
Better access to government services		2
Better chance of receiving RDP housing		3
Close to employment		4
Close to town		5
Close to water		6
Close to electricity		7
Close to sanitation		8
Safety/security reasons		9
Availability of land		10
Close to transport		11
Have friends/relatives/family here		12
Poor opportunities/options elsewhere		13
Living costs are cheap here		14
Close to Clinics/Schools		15
Other (specify) ►		16

7.4. What type of dwelling did this household live in before coming to this settlement?

Dwelling/house or brick/concrete block structure on a separate stand or yard or on farm	1
Traditional dwelling/hut/structure made of traditional materials (Wattle & Daub/mud)	2
Double-storey dwelling	3
Dwelling/house/flat/room in backyard	4
Shack (plastic/semi-permanent material/corrugated iron/cardboard)	5
Shipping Containers	6
Caravan/Tent	7
Other, Specify	8

7.5. Where was this dwelling?

Urban	1
Rural	2
Traditional/Village	3
Farm	4
I don't know	8

7.6. In which region?

Another city in this province	1
Another city in another province	2
Another town in this province	3
Another town in another province	4
Another country	5
Other (Specify) ________________________________	9

7.7. Are you planning, expecting or hoping to move out of the informal settlement within the next 24 months?

Yes, definitely	1
Yes, probably	2
No	3

7.8. Have you, or any member of this household ever applied for a housing subsidy?

Yes	1
No **(Skip to Q7.11)**	2
Do not know **(Skip to Q7.11)**	99

7.9. In what year and month did you apply for a housing subsidy? If respondent does know ENTER 9999 for year and 99 for month

YEAR				MONTH	

7.10. Has your application been successful?

Yes	1
No	2
Application Pending	3
I don't know	4

7.11. Does your household share this dwelling/stand/yard with another household?

Yes	1
No	2
Do not know	99

7.12. What type of occupation rights do you have to the site where your dwelling stands?

Owned and fully paid off **(Go to Q7.15)**	1
Owned but not yet paid off **(Skip to Q7.15)**	2
Rented **(Continue with Q7.13)**	3
Occupied rent-free **(Skip to Q7.18)**	4
Permission to occupy from chief **(Skip to Q7.19)**	5
Recognition from the city **(Skip to Q7.18)**	6
Other	7

7.13. For those who rent, how much is the monthly rent? R___________________

7.14. What kind of rental agreement do you have with the owner?

Written agreement **(Go to Q7.19)**	1
Verbal agreement **(Go to Q7.19)**	2
Informal/Unstructured agreement **(Go to Q7.19)**	3
Other (specify) **(Go to Q7.19)**	4

7.15. For those who have bought their sites, what was the purchase price?

R

7.16. Did you receive any documentation that shows that you have the right to occupy the site where your dwelling stands??

Yes	1
No **(Go to Q7.19)**	2
Do not know **(Go to Q7.19)**	99

7.17. What type of ownership document do you have?

Temporary occupation license	1
Share certificate	2
Title deed	3
Letter from the chief	4
Letter from municipality	5
No document **(Go to Q7.19)**	6
Other (specify) ►	7

7.18. Which year did you receive the ownership/occupation rights? If respondent does not know ENTER 9999 for year.

YEAR			

7.19. Do you have any paying tenants (including sub-letting)?

Yes	1
No **(Skip to Q7.22)**	2

7.20. How many paying tenants do you have (if one person pays rent for a household or group, count the entire group)? NUMBER____________

7.21. How much rent do you receive from these tenants in total each month? R______________

7.22. Did the household undertake any form of improvements to the dwelling in the last 12 months (this EXCLUDES improvements through GOVERNMENT upgrading)?

Yes	1
No **(IF NO, SKIP TO Q7.26)**	2

7.23. Indicate improvement(s) made to the house:

Roof	1
Floor	2
Walls	3
Bathroom	4
Toilet	5
Doors/Windows	6
Added rooms	7
Burglar Doors	8
Other (Specify) ▶	9

7.24. If OPTION 7 in Q7.23 was indicated, how many rooms did you add to your dwelling? NUMBER: _______

7.25. How much did the household spend on improvements in total in the last 12 months? R_________________

7.26. If you want to improve your current dwelling where would you borrow financing from?

Government housing subsidy	1
Micro-credit institution	2
Stockvel	3
Mashonisa	4
Formal Bank	5
Hardware store savings or credit	6
Do not want to borrow money/improve dwelling	7
Other (Specify) ▶	8

7.27. For those who bought/own the dwelling. Would you sell this property if the opportunity arose?

Yes	1
No **(Go to Q7.31)**	2
Not Applicable **(Go to Q7.31)**	9

7.28. If YES to Q7.27, how much do you think you could sell it for?
R__________________

7.29. Would you rent out this property if the opportunity arose?

Yes	1
No **(If NO GO to Q7.31)**	2
Not Applicable **(If Not Applicable, go to Q7.31)**	9

7.30. If YES how much do you think you could rent it for (per month)?
R__________________

7.31. In this community are there any obstacles to you owning land and housing?

Yes	1
No **(Go to Q7.33)**	2
Do not know **(Go to Q7.33)**	8

7.32. If yes, what is the main obstacle? (Circle ONE)

Income	1
Religion	2
Nationality	3
Tribe/Language	4
Health status	5
Constant threat of eviction	6
Gender	7
Outsider	8
Other (specify) ▶	9

7.33. In the past 5 years are you aware of any attempts to evict residents from this settlement?

Yes	1
No	2
Do not know	99

7.34. Have there been any attempts by the municipality to relocate residents of this settlement to another area?

Yes	1
No	2
Do not know	99

7.35. Are you aware of municipal rules (by-laws) which apply to residential areas?

Yes, I am aware of most	1
Yes, I am aware of some	2
Yes, I am aware of a few	3
No, I am not aware of by-laws **(Go to 7.37)**	4

7.36. Do municipal rules (by-laws) apply to this settlement (e.g. slaughtering of cows/sheep, setting up businesses/factories, etc.)?

Yes	1
No	2
Do not know	99

7.37. Do you know if your area has been approved for settlement by the municipality?

Yes	1
No	2
Do not know	99

MODULE 8: INFRASTRUCTURE AND SERVICE DELIVERY

8.1. In the last year, have you had any problems with noise from any of these sources (INTERVIEWER: READ OUT ALL THE SOURCES FOR RESPONDENT TO ANSWER)

	Yes	No	Some-times	Do not know
a. Immediate neighbours	1	2	3	8
b. People in your street	1	2	3	8
c. Road traffic – cars, buses, motorbikes or lorries	1	2	3	8
d. Trains	1	2	3	8
e. Aeroplanes	1	2	3	8
f. Car alarms or burglar alarms	1	2	3	8
g. Factories or workshops	1	2	3	8
h. Building sites	1	2	3	8
i. Road works	1	2	3	8
j. Pubs, clubs, or entertainment, shebeens	1	2	3	8
k. Animals e.g. dogs, cats	1	2	3	8
l. Other (Specify) ►	1	2	3	8
m. None of these (spontaneously only)	1	2	3	8

If your answer to Q8.1 (a) above was YES, ASK Q8.2

8.2. Do you think that the problems you have with the immediate neighbours are mainly because of the type of dwellings or the behaviour of people who live there?

Design of the building	1
Behaviour of the people who live there	2
Both equally	3
Neither	4
Don't know (spontaneous only)	9

WATER

8.3. What is the household's MAIN source of drinking water?

Piped tap water in dwelling	1
Piped tap water on site/yard	2
Public/communal tap	3
Neighbour	4
Water carrier/ tanker	5
Water carrier/tank on site	6
Borehole on site	7
Borehole outside yard	8
Rainwater tank on site	9
Flowing water/stream/spring	10
Stagnant water/Dam/pool	11
Well	12
Spring	13
Other (Specify) ►	14

8.4. How far is the water source from the dwelling or yard (200 m is equal to the length of two football/soccer fields)? (Fieldworker to observe)

Water source is in dwelling	1
Less than 200 metres	2
201 - 500 metres	3
501 metres - 1 kilometre	4
More than 1 kilometre	5
Don't Know	6

8.5. Is the water from the main source of drinking water before any treatment… [ask all options below]

	1 = Yes	2 = No
1 = Safe to drink?	1	2
2 = Clear (has no colour / free of mud)?	1	2
3 = Good in taste?	1	2
4 = Free from bad smells?	1	2

8.6. Do household members treat the water used for drinking? This may include boiling, adding chlorine or other chemicals, filtering.

Yes, always	1
Yes, sometimes	2
No, never **(Go to Q8.14)**	3

8.7. What method do you use to treat the water you drink?

Boiling	1
Natural Filtering	2
Chemical (e.g. chlorine)	3
Other (Specify) ►	4

8.8. Is your main source of drinking water supplied by a municipality?

Yes	1
No **(Go to Q8.14)**	2
Don't Know **(Go to Q8.14)**	3

8.9. Does the household pay for municipal water? If cost of water is included in a levy/rent paid to a housing complex/owner/landlord, the response should be "No"

Yes **(Go to Q8.11)**	1
No	2
Don't Know **(Go to Q8.11)**	3

Ask if "No" in Q8.9

8.10. What is the main reason why the household does not pay for water

Use own source of water	1
Use a free water source	2
Pay directly to landlord as part of rent	3
Payment included in levy	4
Permission from municipality not to pay	5
Do not have water meter	6
Water meter not working/broken	7
Do not receive water bill	8
Community decision not to pay	9
Cannot afford to pay	10
Water supply irregular	11
Water supply has been stopped	12
Other (specify)	13

8.11. Has your municipal water supply been interrupted at any time during the last 12 months?

Yes	1
No **(Go to Q8.14)**	2

Ask if 'Yes' in Q8.11

8.12. If yes, what was the main reason for the interruption?

Use own source of water	1
Use a free water source	2
Pay directly to landlord as part of rent	3
Payment included in levy	4
Permission from municipality not to pay	5
Do not have water meter	6
Water meter not working/broken	7
Do not receive water bill	8
Community decision not to pay	9
Cannot afford to pay	10

8.13. Thinking about the interruptions in your municipal water supply over the last 12 months, was any specific interruption longer than two days?

Yes	1
No	2
Don't Know	3

SANITATION

8.14. What kind of toilet facility does your household use?

Flush toilet connected to a municipal sewage system	1
Flush toilet connected to a septic tank	2
Chemical toilet	3
Pit latrine with ventilation pipe (long drop)	4
Pit latrine without ventilation pipe (long drop)	5
Bucket toilet	6
Nearby veld	7
Other, specify	8
None	9

8.15. Do you share this toilet facility with other households?

Yes	1
No	2

8.16. How many people share this toilet facility? NUMBER: ☐

8.17. Where is the toilet facility located?

In dwelling	1
On site (in yard)	2
Off site (outside yard)	3

8.18. During the past 6 months, have you experienced any of the following problems with regards to the toilet facility usually used by this household? *Ask all options below.*

	Yes	No	N/A
No water to flush the toilet	1	2	9
Toilet blocked up	1	2	9
Toilet pit or chamber full	1	2	9
Toilets not well maintained and broken	1	2	9
Poor lighting	1	2	9
Unsafe to use the toilet, due to risk of assault	1	2	9
Toilet unsafe to use, due to health risks	1	2	9
Toilet not enclosed well or structure damaged	1	2	9
Broken pipes or blockages in the municipal system	1	2	9
Too many people, long waiting times	1	2	9
No tap or water point to wash hands after using the toilet	1	2	9
Problem reported but not repaired within 5 working days	1	2	9

8.19. Does your unit have a bathroom/shower?

Yes	1
No **(Go to Q8.22)**	2

8.20. Is your bathroom/shower shared with another household?

Yes	1
No **(Go to Q8.22)**	2

8.21. IF YES, how many people do you share this shower/bathroom with?
NUMBER: : ☐

REFUSE REMOVAL

8.22. Is rubbish or litter lying around a problem in this area?

A serious problem in this area	1
A problem in this area but not serious	2
Not a problem in this area	3
Not aware	8

8.23. How does your household dispose of rubbish/garbage?

Removed by local authority/private company at least once a week	1
Removed by local authority/private company less often than once a week	2
Removed by community members, contracted by the Municipality, at least once a week	3
Removed by community members, contracted by the Municipality, less often than once a week	4
Removed by community members at least once a week	5
Removed by community members less often than once a week	6
Communal refuse dump/communal container	7
Own refuse dump	8
Dump or leave rubbish anywhere (e.g. roadside, river/pond etc)	9
Burn it (e.g. in communal pit, in the open, etc)	10
Bury it	11
It is recycled	12
Other (specify)	13

SOURCES OF LIGHTING AND HEATING

8.24. Does this household have access to/use electricity?

Yes	1
No **(Go to Q8.28)**	2
Don't Know **(Go to Q8.28)**	3

8.25. How is the electricity connection made?

Metered connection to the house (In-house meter)	1
Connection from neighbour's house	2
Connection from street	3
Other (specify) ______________________________	4

8.26. How many blackouts/electricity outages do you experience per week? If none ENTER 0. NUMBER: ______

8.27. How many hours per day do you get electricity? HOURS: ______

8.28. What is the main source of energy for (a) HEATING, (b) COOKING and (c) LIGHTING in this household?

	**(a) Main source for HEATING [*Circle one option*]**	**(b) Main source for COOKING [*Circle one option*]**	**(c) Main source for LIGHTING [Circle one option]**
a. Paraffin	1	1	1
b. Candle	2		2
c. Solar System	3	3	3
d. Electricity	4	4	4
e. Batteries	5	5	5
f. Car batteries	6	6	6
g. Generator (petrol/diesel)	7	7	7
h. Coal	8	8	
i. Gas	9	9	
j.. Warm clothing	10		
k. Hot water bottle	11		
l. Blankets (not electric)	12		
m. Wood	13	13	13
n. Other (specify) ►	14	14	14

8.29. Are the following adequate or inadequate for your household's needs? (This question applies regardless of energy source)

	**Adequate**	**Not adequate**
1. The amount of energy for lighting	1	2
2. The amount of energy for cooking	1	2
3. The amount of energy for heating rooms and keeping warm	1	2

8.30. If inadequate, what is the main reason why you feel that the amount of energy is inadequate to meet your household's needs? (*INTERVIEWER*: CIRCLE ONE OPTION ONLY)

Not enough money to pay for the energy we need	1
There are many electricity power cuts in my area	2
The supply of electricity to my household is limited	3
Firewood is very scarce	4
Gas or paraffin not always available in the shops	5
Other (specify) ►	8
(Don't know/Uncertain)	99

ACCESS TO SERVICES

For the next service points; indicate how you get there and how much time and/or money you spend (one-way trip)? *INTERVIEWER: THIS IS A MULTIPLE RESPONSE QUESTION*

		**8.31. Mode of Transport**<br>01 = Walk<br>02 = Bicycle<br>03 = Private vehicle<br>04= Bus<br>05 = Train<br>06 = Taxi<br>07 = Animal/Cart<br>08 = Other (Specify)	**8.32. Time spent travelling (INSTRUCTION TO *INTERVIEWER*: Please enter time in minutes only e.g. instead of 1 hour 30 mins ENTER 90): If respondent does not know enter 999**	**8.33. Cost of travelling**<br>(***INSTRUCTION TO INTERVIEWER***: *Please enter RAND amount of the FARE rounded off to the nearest RAND* ***e.g. instead of R10.30 ENTER 10 and R10.50 ENTER 11****):*<br>*If respondent does not know cost of using own vehicle enter 7777*<br>*If respondent is walking/cycling enter 8888*<br>*If respondent does not know enter 9999*
1	Government post office			R
2	Government clinic			R
3	Government hospital			R
4	Police station			R
5	School			R
6	Municipal offices			R
7	Home Affairs offices where one can register births and deaths			R
8	Taxi rank			R
9	Bus station			R
10	Train station			R
11	Bank			R
12	Access to money point e.g. ATM, Mpesa, Money market			R
13	Work			R
14	Shops			R
15	To the city			R

8.34. Are emergency services able to access this settlement without difficulty?

Yes	1
No	2

8.35. If you need emergency services in this settlement (GIVE OPTIONS), are they readily available?

	**Available**	**Sometimes**	**Not Available**
a. Ambulances	1	2	3
b. Fire Brigade	1	2	3
c. Police	1	2	3

8.36. In your view, in the last one year, have employment opportunities increased, decreased or remained the same amongst people in your settlement?

a. Increased	1
b. Decreased	2
c. Stayed the same	3

8.37. What are the environmental challenges in this settlement? (Tick all that apply)

a. Smog	1
b. Air pollution	2
c. Water pollution	3
d. Uncollected waste/litter	4
e. Noise pollution (e.g. traffic)	5
f. Lack of vegetation	6

8.38. While living in this settlement, have you experienced the following?

	Yes	No
a. Fire in the dwelling	1	2
b. Flooding	1	2
c. Mudslides	1	2

IF 'YES' TO A), CONTINUE TO Q8.39
IF 'YES' TO B), SKIP TO Q8.40
IF NO to all, SKIP TO Q8.41

8.39. What was the cause of the fire?

a. Illegal electricity Connection	1
b. Use of paraffin/gas stove	2
c. Candles	3
d. Cigarettes	4
e. Arson (Deliberate burning)*	5
f. Other (specify) ▶	6

NB: Arson is the criminal act of deliberately setting fire to property.

8.40. What was the cause of the flood?

a. Storm	1
b. Poor/Lack of drainage in the area	2
c. Settlement in flood plain	3
d. Other (specify) ▶	4

8.41. Have there been any attempts to reduce the following environmental challenges in the settlement?

	Yes	No	N/A
a. Smog	1	2	3
b. Soil Erosion	1	2	3
c. Water pollution	1	2	3
d. Dumping of solid waste	1	2	3
e. Noise pollution (e.g. traffic)	1	2	3
f. Flooding	1	2	3
g. Fires	1	2	3
h. Strong winds	1	2	3

8.42. Are you aware of any programmes in this settlement that deal with the following?

	Yes	No	N/A
a. Health education	1	2	3
b. Fire prevention (specify structural/veld)	1	2	3
c. Disaster management	1	2	3
d. Recycling	1	2	3
e. Prevention of soil erosion	1	2	3
f. Waste management	1	2	3
g. Planting of trees	1	2	3
h. Environmental education/preservation	1	2	3
i. Wind Breaks	1	2	3

MODULE 9: SATISFACTION

INTERVIEWER READ OUT: Now I would like to ask you some questions about how satisfied you are with your local area.

I am going to read out a list of things that can cause problems for people in their area. I would like you to tell me whether, in your opinion, each of them is a problem in this area. They might not affect you personally but you may feel that such things are general problems in this area.

9.1. How satisfied or dissatisfied are you with the following?

Item	Very Satisfied	Satisfied	Neither satisfied nor dissatisfied	Dissatisfied	Very dissatisfied	I Don't Know
Household water quality	1	2	3	4	5	8
Supply of water	1	2	3	4	5	8
Sanitation services	1	2	3	4	5	8
Frequency of electricity supply	1	2	3	4	5	8
Wastewater (i.e. dirty water) collection service	1	2	3	4	5	8
Refuse removal	1	2	3	4	5	8
Public service delivery in general	1	2	3	4	5	8
Public transport links	1	2	3	4	5	8
Police service in your settlement	1	2	3	4	5	8
Employment opportunities	1	2	3	4	5	8
Support from your community	1	2	3	4	5	8
Housing	1	2	3	4	5	8
Land issues (ownership)	1	2	3	4	5	8

9.2. How would you describe the quality of the relationship between this community and local government?

Very good	1
Good	2
Neither good nor bad	3
Bad	4
Very bad	5

9.3. Do you feel that the municipality is responsive to the needs of this community?

Very Responsive	1
Moderately Responsive	2
Rarely Responsive	3
Not Responsive	4

MODULE 10: SOCIAL CAPITAL, SOCIAL NETWORKS AND COMMUNITY PARTICIPATION

10.1. How important is it for you to help people whether by sharing time, money, or possessions?

An important part of my life	1
Not an important part of my life	2
Neither important nor unimportant	3
Do not know	9

10.2. Do you or any other member of this household get help from anyone?

Yes	1
No **(SKIP TO Q10.6)**	2
Do not know **(SKIP TO Q10.6)**	9

10.3. What sort of help do you or your household members get?

Money	1
Groceries/ food	2
Clothes	3
Child minding	4
Other	5
Refused to answer	8
Do not know	9

10.4. Who provides the help? (MULTIPLE OPTIONS)

Neighbours	1
Family	2
Non-governmental organisation	3
Government	4
Church/religious group	5
Other	6
Refused to answer	8
Do not know	9

10.5. Are there people WHO HELP YOU in your community?

Yes	1
No	2
Refused to answer	8
Do not know	9

10.6. Do you or any of your household members give help or money or goods to anyone?

Yes	1
No **(SKIP TO Q10.9)**	2
Do not know **(SKIP TO Q10.9)**	8

10.7. What type of help do you or your household members give?

Money	1
Groceries/ food	2
Clothes	3
Child minding	4
Other	5
Do not know	8
Refused to answer	9

10.8. To whom do you or your household members give help?

Family	1
Neighbours	2
Relatives	3
Non-governmental organization	4
Church/religious group	5
Other (specify) ►	6
Do not know	8
Refused to answer	9

10.9. Are there people YOU help in your community?

Yes	1
No	2
Do not know	8
Refused to answer	9

10.10. Do you expect (immediately or in future) that if you help someone in your community, they should also help you in return?

Yes	1
No	2

10.11. To what extent would you agree that people in this area generally treat each other with respect in public?

Strongly agree	1
Agree	2
Neither agree nor disagree	3
Disagree	4
Strongly disagree	5
Do not know	9

10.12. How would you rate the community spirit (togetherness) in your settlement?

Very Good	1
Good	2
Average	3
Poor	4
Very Poor	5

**COMMUNITY INVOLVEMENT**	**10.13. Do you have [OPTION] active in your settlement?**			**10.14. Does anyone from this household participate in [OPTION]?**			**10.15. Did they attend any meetings of the [OPTION] in the past 12 months?**		
	**Yes**	**No**	**Don't Know**	**Yes**	**No**	**Don't Know**	**Yes**	**No**	**Don't Know**
a. A neighbourhood improvement group	1	2	3	1	2	3	1	2	3
b. Health volunteers (e.g. Home Based Community care, HIV Forum etc)	1	2	3	1	2	3	1	2	3
c. A sports club	1	2	3	1	2	3	1	2	3
d. A neighbourhood security watch organisation	1	2	3	1	2	3	1	2	3
e. Local national political party	1	2	3	1	2	3	1	2	3
f. Religious organisations	1	2	3	1	2	3	1	2	3
g. Parent-teacher associations	1	2	3	1	2	3	1	2	3
h. Stockvel Group	1	2	3	1	2	3	1	2	3
i. Burial Society	1	2	3	1	2	3	1	2	3
j. Resident Association	1	2	3	1	2	3	1	2	3
k. Other (specify) ►	1	2	3	1	2	3	1	2	3

10.16. Which of the following fits your situation?

a. I know most of the people in the group(s) I am involved in	1
b. I know a few people in my group, but most are strangers in the group(s) I am involved in	2

10.17. Would you contact members of this group if:

	Yes	No
a. You needed to get things done (e.g. find a baby sitter, find a school, get financial advice or other similar help?)	1	2
b. You needed a job?	1	2
c. You were upset or 'in trouble' and needed personal help and support?	1	2

10.18. Does anyone in the group come to you for assistance?

Yes	1
No	2

10.19. Are you aware that this settlement is targeted for improvement?

Yes	1
No **(Go to Q10.32)**	2
Do Not Know **(Go to Q10.32)**	

10.20. How did you find out?

d. Family/Friends/Neighbours in the settlement	1
e. Resident Association	2
f. The group(s) I am involved in	3
g. Municipality	4

10.21. If your answer to Q10.20.C was 'Yes', which group(s) was it?

a. A neighbourhood improvement group	1
b. Health volunteers	2
c. A sports club	3
d. A neighbourhood security watch organisation	4
e. Local politics	5
f. Religious organisations	6
g. Parent-teacher associations	7
h. Stokvel Group	8
i. Burial Society	9
j. Other (specify) ▶	10

10.22. Which agencies are involved in the improvement of this settlement?

	**YES**	**NO**	**I Don't know**
a. NGO/CBO	1	2	9
b. Residence Association	1	2	9
c. Private Developers	1	2	9
d. Contractors	1	2	9
e. Municipality	1	2	9

10.23. Does a community structure exist that involves the residents in the improvement of this settlement? What type of structure exists?

	**YES**	**NO**
a. Steering committee	1	2
b. Community Representatives	1	2
c. Residence Association	1	2
d. Community Trust	1	2
e. Community Based Organisations	1	2
f. Community Development Forum	1	2
g. SA National Civic Organisation (SANCO)	1	2
h. Other (specify) ▶	1	2

10.24. If a community structure exists, do they keep minutes of their meetings?

Yes	1
No	2
Sometimes	3
Do Not Know	4

10.25. Has the community signed a social agreement for improvement of this settlement with the municipality or any other agencies involved in the improvement of this settlement?

Yes	1
No	2
Do Not Know	3

10.26. Are you aware of a plan to involve the community in improving this settlement?

Yes	1
No	2
Do Not Know	3

10.27. Has any member of your household been involved in conducting the community survey required for improving this settlement?

Yes	1
No	2
Do Not Know	8

10.28. Are any of the following members employed in improving this settlement? (e.g. building roads, providing water and sanitation, building the dwellings)

	Yes	No
a. Household members	1	2
b. Community members	1	2

10.29. Are you aware of any training that was conducted for members of this settlement to participate improving this settlement?

Yes	1
No **(Go to Q10.31)**	2
Do Not Know **(Go to Q10.31)**	3

10.30. Who provided the training?

a. Municipality	1
b. NGOs	2
c. CBOs	3
d. Community Leadership Structures	4
e. Developer	5
f. Don't Know	6
g. Other (Specify) ►	7

10.31. Did the community have a say on the following in the upgrading processes:

	Yes	No	I Don't Know
a. Level of water services	1	2	8
b. Level/Type of toilets	1	2	8
c. Provision of electricity	1	2	8
d. Type of roads (e.g. gravel, tarmac)	1	2	8
e. Size of dwellings	1	2	8
f. Type of building materials	1	2	8
g. Level of basic social services (schools, clinics, police station)	1	2	8
h. Multipurpose Hall	1	2	8

10.32. In this community, has any land been allocated for the following?

	Yes	No	I Don't Know	N/A
a. Social Services (Schools, Clinics, Shops, Police Station, Postal Services, etc)	1	2	8	9
b. Library	1	2	8	9
c. Multipurpose Hall	1	2	8	9
d. Government-related mobile services	1	2	8	9
e. Playgrounds/Parks	1	2	8	9

10.33. While in this settlement, have you or any member of the household done any of the following? (Ask respondent all options whilst taking into account SKIP patterns)

ACTIVITY	Yes	No
1. Voted in local government elections	1	2
2. Contacted your elected representative	1	2
3. Contacted newspapers, radio, or TV to generate interest in a problem	1	2
4. Actively participated in an information campaign (e.g. HIV awareness etc.)	1	2
5. Participated in a protest (e.g. service delivery)	1	2

If No to option 1, continue
If yes to option 5, ask Q35 If NO go to Q10.36

10.34. If NO to Q10.33.1, above. What is the MAIN reason why you did not vote? Ask the respondent to select ONLY ONE MAIN reason for not voting.

a.	Whether I vote or not makes no difference	1
b.	I did not register	2
c.	I am not a citizen	3
d.	Fear of political intimidation	4
e.	Other (Specify) ►	8

10.35. Ask if YES to Q10.33.5. What was the protest about?

a.	Housing	1
b.	Water	2
c.	Sanitation	3
d.	Electricity	4
e.	Schools	5
f.	Health Services	6
g.	Protests against foreign nationals	7
h.	All of the above	8
i.	Other (Specify) ►	99

10.36. Ask if NO to Q10.33.5. Have there been protests in this settlement?

Yes **(Go to Q10.37)**	1
No **(If NO GO to Q10.40)**	2
Do not know	99

10.37. Ask if YES to Q10.36. What was the protest about?

a.	Housing	1
b.	Water	2
c.	Sanitation	3
d.	Electricity	4
e.	Schools	5
f.	Health Services	6
g.	Protests against foreign nationals	7
h.	All of the above	8
i.	Other (Specify) ►	99

10.38. Did the protest(s) lead to violence or the destruction of property?

Yes	1
No	2
Do not know	9

10.39. What forms of violence and harassment exist in your community?

Organised	1
Non-organised	2
None	3

10.40. Would you describe yourself as being a member of a group that is discriminated against in this community?

Yes	1
No **(IF NO, SKIP TO Q10.44)**	2

10.41. On what grounds is your group discriminated against? (INTERVIEWER: Circle ALL THAT APPLY)

a.	Colour/race	1
b.	Tribe/ ethnicity	2
c.	Religion	3
d.	Language	4
e.	Age	5
f.	Gender	6
g.	Sexual orientation	7
h.	Education	8
i.	Disability	9
j.	Unemployment	10
k.	Region/province	11
l.	Nationality	12
m.	Other, Specify	13
n.	Do not know	99

10.42. Where has this discrimination happened to you most recently? (INTERVIEWER: TICK ALL THAT APPLY)

At work	1
At an educational institution	2
In shops	3
At church	4
On the road or street	5
When applying for a job	6
In a government department	7
In social clubs	8
In theatres	9
In restaurants	10
In sport	11
Other (specify) ▶	12

10.43. How often do you feel that members of your group are discriminated against?

Always	1
Often	2
Sometimes	3

10.44. In what way do you participate in resolving the needs of this community?

	Yes	No
**1.** Attend ward meetings/ward committee	1	2
**2.** Service delivery protest	1	2
**3.** Make a written submission during the IDP consultation process	1	2
**4.** Contact the regional offices	1	2
**5.** Petition the city	1	2
**6.** Raise the issue during the mayoral road show, the imbizo	1	2
**7.** Speak to my ward councillor	1	2
**8.** Speak out in the media	1	2
**9.** Resident Association	1	2
**10.** Not Interested **(Go to Q10.45)**	1	2
**11.** Other (specify) ▶	1	2

10.45. If YES to Q10.44.10 ASK. If not interested, what are the reasons for not being interested in participating?

Do not have time	1
Venues not suitable	2
Time is not suitable	3
Authorities don't take participation seriously	4
There are community organisations that do these things	5
I don't care	6
Other (specify) ▶	7

10.46. Which structures represent the interests and demands of residents in this community?

SA National Civic Organisation (SANCO)	1
Homeless people's Federation	2
Church groups	3
Trade Unions	4
Political parties	5
Ward committee	6
Residents Association	7
Community Development Forum	8
Other (specify…)	9

10.47. What are the most important issues that are discussed during public consultation meetings in this settlement? (INTERVIEWER: ALL THAT APPLY)

Housing	1
Water	2
Sanitation	3
Street lighting	4
Community safety	5
Public transport	6
Tarred roads	7
Unemployment	8
Do not know	9

MODULE 11: CRIME AND SAFETY

11.1. How would you describe the level of crime in this area?

Crime is a serious problem in this area	1
Crime is a problem in this area but not serious	2
Crime is not a problem in this area at all	3
I don't know	4

11.2. How safe do you feel against criminals in this <u>SETTLEMENT</u>?

Not safe	1
Fairly safe	2
Safe	3
Very safe	4

11.3. Is it safe for women and children to walk around by themselves during the day in this settlement?

Not safe	1
Fairly safe	2
Safe	3
Very safe	4

11.4. Is there any form of gender based violence where women are specifically targeted?

Yes	1
No	2

11.5. Has mob justice/violence occurred within the settlement in the past 12 months?

Yes	1
No	2

11.6. How safe do you feel against criminals in your own <u>HOME</u>?

Not safe	1
Fairly safe	2
Safe	3
Very safe	4

11.7. Has your household been broken into in the last 12 months?

Yes	1
No	2

11.8. In the past 12 months, was this household a victim of arson? *(Arson is the criminal act of deliberately setting fire to property)*

Yes	1
No	2

11.9. In the past 12 months was any member of this household murdered?

Yes	1
No	2

11.10. In the past 12 months, has anybody in this household been a victim of crime (excluding house burglaries)?

Yes	1
No **(Go to Q11.16)**	2

PERSON CODE	11.11. What type of crime was it?<br><br>1 = Theft<br>2 = Attempted Murder<br>3 = Extortion (Bribery, Blackmail)<br>4 = Gun injury<br>5 = Hijacking<br>6 = Knife injury<br>7 = Mugging<br>8 = Rape<br>9 = Severe beating<br>10 = = Other (Specify)	11.12. Who was the perpetrator?<br><br>1 = Household Member<br>2 = Neighbour<br>3 = Gang member, same settlement<br>4 = Gang member, outside settlement<br>5 = Employer<br>6 = Police<br>7 = Unknown Perpetrator<br>8 = Other (Specify)	11.13. Where did it occur?<br><br>1 = At home<br>2 = In the settlement<br>3 = In a neighbouring settlement<br>4 = Elsewhere (Specify)_____________
1			
2			
3			
4			
5			
6			
7			
8			
9			
10			
11			
12			
13			
14			
15			
16			
17			
18			

11.14. Have you reported any of these incidents to the police?

Yes	1
No	2

11.15. Whether you reported or did not report to the police, did you do anything else to deal with the crime?

Yes	1
No	2

11.16. Is your community doing anything to reduce crime?

Yes	1
No	2

11.17. In the last one year, has crime increased, decreased or stayed the same in this settlement?

Increased	1
Decreased	2
Stayed the same	3

11.18. Do you trust the police to effectively reduce crime in the area?

Yes	1
No	2

11.19. How responsive are police to issues related to crime in this settlement?

Very Responsive	1
Moderately Responsive	2
Rarely Responsive	3
Not Responsive	4

MODULE 12: ATTITUDES TOWARDS FOREIGNERS

INTERVIEWER: *This section is assessing attitudes and perceptions towards foreigners in general, as such there is no need to identify specific nationalities. Ask all respondents.*

12.1. If respondent is South African ASK. Do you have any friends who are foreigners?
If respondent is a foreign national ASK. Do you have any friends who are South African?

Yes	1
No	2
Refused to answer	9

12.2. How would you rate the attitude of people in this settlement towards foreigners?

Very Friendly	1
Friendly	2
Neither Hostile Nor Friendly	3
Hostile	4
Very Hostile	5

12.3. Compared to 3 years ago, would you say relationships between locals and foreigners in this settlement have….?

Greatly improved	1
Improved	2
Remained the same	3
Become worse	4
Greatly deteriorated	5

12.4. In the last 3 years, would you say the number of foreigners in this settlement has…

Greatly increased	1
Increased	2
Remained the same	3
Reduced	4
Greatly reduced	5

12.5. How do attitudes towards foreigners manifest themselves in this community?

	**Yes**	**No**
1. Through the use of derogatory terms such as referring to foreigners as makwerekwere	1	2
2. Propaganda against *foreigners (Propaganda is the deliberate, systematic attempt to shape perceptions, and direct behaviour to achieve a response that furthers the desired intent of the propagandist)*	1	2
3. Hate speech against foreigners	1	2
4. Violence against foreigners	1	2
5. Looting & destruction of shops & businesses that belong to foreigner	1	2

**12.6. Kindly indicate whether you agree or disagree with the following statements…**	Strongly disagree	Disagree	Neither agree nor disagree	Agree	Strongly agree	(Do not know)
1. Foreigners benefit from RDP houses	1	2	3	4	5	8
2. Foreigners do not contribute to the economy	1	2	3	4	5	8
3. Foreigners are stealing our jobs	1	2	3	4	5	8
4. Foreigners in my settlement do not have legal documentation	1	2	3	4	5	8
5. Foreigners in my settlement are involved in illegal activities	1	2	3	4	5	8

12.7. Do you agree with the following statement… foreigners in my settlement should be sent back to their home countries

Yes	1
No	2
Refused to answer	9

THANK YOU FOR YOUR COOPERATION

FIELDWORKER	FIELDWORK CHECKER	TEAM LEADER	PROVINCIAL COORDINATOR
NAME:	NAME:	NAME:	NAME:
NUMBER:	NUMBER:	NUMBER:	NUMBER:

KISH GRID

QUESTIONNAIRE NUMBER				NUMBER OF HOUSEHOLDS ON STAND / SITE																								
				**1**	**2**	**3**	**4**	**5**	**6**	**7**	**8**	**9**	**10**	**11**	**12**	**13**	**14**	**15**	**16**	**17**	**18**	**19**	**20**	**21**	**22**	**23**	**24**	**25**
**1**	**26**	**51**	**76**	1	1	1	3	2	4	1	3	5	8	6	5	12	10	1	6	8	7	19	19	13	21	13	24	25
**2**	**27**	**52**	**77**	1	2	3	4	3	1	2	2	3	4	8	3	7	2	5	14	4	15	4	8	6	16	14	22	19
**3**	**28**	**53**	**78**	1	1	2	1	4	2	7	6	9	3	5	11	2	1	3	11	7	10	16	16	10	5	2	2	3
**4**	**29**	**54**	**79**	1	2	3	2	1	3	5	8	6	2	4	2	4	8	11	10	16	6	9	10	15	11	12	11	18
**5**	**30**	**55**	**80**	1	1	1	4	5	6	3	5	7	5	9	8	13	3	2	13	5	18	1	4	1	20	11	5	24
**6**	**31**	**56**	**81**	1	2	2	2	3	5	7	7	8	7	1	4	9	14	8	2	17	17	14	12	14	22	10	3	14
**7**	**32**	**57**	**82**	1	2	1	1	4	1	4	1	4	6	3	6	5	7	13	9	2	3	13	14	8	2	7	20	4
**8**	**33**	**58**	**83**	1	1	2	3	2	5	1	4	2	1	7	10	6	5	4	15	10	5	2	13	4	17	5	17	8
**9**	**34**	**59**	**84**	1	1	3	2	5	6	2	2	1	9	10	1	10	4	6	6	1	9	10	1	5	6	9	1	12
**10**	**35**	**60**	**85**	1	2	2	4	1	3	3	6	9	10	11	12	3	9	15	7	8	11	6	3	9	4	3	10	1
**11**	**36**	**61**	**86**	1	1	1	3	1	4	5	3	1	6	2	9	13	11	14	4	11	4	15	15	17	1	1	23	2
**12**	**37**	**62**	**87**	1	2	3	1	3	2	7	5	6	5	7	7	8	6	10	3	3	1	12	20	7	13	22	12	16
**13**	**38**	**63**	**88**	1	1	2	1	5	3	6	4	3	4	6	2	11	13	12	1	15	8	7	2	12	15	21	13	7
**14**	**39**	**64**	**89**	1	2	3	2	4	1	4	7	8	2	5	6	11	12	9	16	13	16	11	18	18	14	16	18	23
**15**	**40**	**65**	**90**	1	2	1	4	2	4	3	8	7	7	11	1	3	5	7	12	14	13	8	17	20	19	20	19	11
**16**	**41**	**66**	**91**	1	1	3	3	1	6	5	1	5	9	10	3	2	11	13	8	12	12	5	6	21	8	8	4	15
**17**	**42**	**67**	**92**	1	1	2	2	3	4	2	6	2	3	2	12	5	2	10	13	5	8	18	9	16	10	17	16	20
**18**	**43**	**68**	**93**	1	2	1	4	2	6	4	1	4	8	9	10	7	9	3	12	12	9	7	20	19	9	19	21	13
**19**	**44**	**69**	**94**	1	2	2	1	3	5	2	8	9	10	4	9	8	13	1	1	14	10	19	10	11	18	15	7	6
**20**	**45**	**70**	**95**	1	1	3	2	5	4	1	3	8	1	3	8	6	6	9	5	7	13	4	15	1	7	22	15	21
**21**	**46**	**71**	**96**	1	1	1	2	5	1	7	2	3	2	1	11	4	7	5	3	2	1	3	12	18	5	19	14	9
**22**	**47**	**72**	**97**	1	2	1	3	1	3	2	6	2	1	8	7	1	4	2	11	8	2	17	4	17	21	16	3	5
**23**	**48**	**73**	**98**	1	2	3	4	2	2	6	7	7	8	3	4	9	3	6	2	11	11	16	2	8	11	23	6	22
**24**	**49**	**74**	**99**	1	1	2	1	4	6	3	5	5	3	1	5	13	1	14	8	14	6	15	9	14	3	6	9	17
**25**	**50**	**75**	**100**	1	1	2	3	3	2	4	6	4	7	5	3	12	12	12	4	6	2	17	11	2	12	4	8	10

PAGE INTENTIONALLY LEFT BLANK

PAGE INTENTIONALLY LEFT BLANK

IDENTIFICATION FLAP

	HOUSEHOLD NUMBER						
	QUESTIONNAIRE NUMBER						

	i	ii	iii
**PERSON CODE**	**INTERVIEWER NAME**<br>____________________<br>WRITE DOWN FIRST NAME AND SURNAME OF EACH MEMBER OF THE HOUSEHOLD, **STARTING** WITH THE **HEAD** OR **ACTING HEAD**. IF MORE THAN ONE HEAD OR ACTING HEAD TAKE THE OLDEST.<br><br>***INTERVIEWER ASK:***<br>Has [NAME] lived under this "roof" or within the same compound/homestead/stand at least 15 days during the **LAST 12 MONTHS**<br>**OR** did they **ARRIVE HERE IN THE LAST 15 DAYS** and this is now their usual residence and when they are (were) together you **SHARE/D FOOD FROM A COMMON SOURCE** with other household members and when they are (were) together **YOU CONTRIBUTE/D TO OR SHARE/D IN A COMMON RESOURCE POOL.**	CIRCLE ID OF ALL CHILDREN AGED 0 – 36 MONTHS (3 YEARS)	CIRCLE ID OF ALL CHILDREN AGED 36 -60 MONTHS (3 – 5 YEARS)
	**NAME & SURNAME OF HOUSEHOLD MEMBER**		
**1**	NAME & SURNAME OF HEAD OF HOUSEHOLD	**1**	**1**
**2**		**2**	**2**
**3**		**3**	**3**
**4**		**4**	**4**
**5**		**5**	**5**
**6**		**6**	**6**
**7**		**7**	**7**
**8**		**8**	**8**
**9**		**9**	**9**
**10**		**10**	**10**
**11**		**11**	**11**
**12**		**12**	**12**
**13**		**13**	**13**
**14**		**14**	**14**
**15**		**15**	**15**
**16**		**16**	**16**
**17**		**17**	**17**
**18**		**18**	**18**
